# *In vivo* fermentation production of humanized noncoding RNAs carrying payload miRNAs for targeted anticancer therapy

**DOI:** 10.7150/thno.56596

**Published:** 2021-03-04

**Authors:** Peng-Cheng Li, Mei-Juan Tu, Pui Yan Ho, Neelu Batra, Michelle M.L. Tran, Jing-Xin Qiu, Theodore Wun, Primo N. Lara, Xiang Hu, Ai-Xi Yu, Ai-Ming Yu

**Affiliations:** 1 Department of Orthopedic Trauma and Microsurgery, Zhongnan Hospital of Wuhan University, Wuhan, Hubei 430071, China.; 2 Department of Biochemistry & Molecular Medicine, UC Davis School of Medicine, Sacramento, CA 95817, USA.; 3 Department of Pathology, Roswell Park Cancer Institute, Buffalo, NY 14263, USA.; 4 Division of Hematology Oncology, UC Davis School of Medicine, Sacramento, CA 95817, USA.; 5 Department of Internal Medicine, UC Davis School of Medicine, Sacramento, CA 95817, USA.

**Keywords:** ncRNA, miRNA, bioengineering, therapy, cancer

## Abstract

**Rationale:** Noncoding RNAs (ncRNAs) such as microRNAs (miRs or miRNAs) play important roles in the control of cellular processes through posttranscriptional gene regulation. However, ncRNA research is limited to utilizing RNA agents synthesized *in vitro*. Recombinant RNAs produced and folded in living cells shall better recapitulate biologic RNAs.

**Methods:** Herein, we developed a novel platform for *in vivo* fermentation production of humanized recombinant ncRNA molecules, namely hBERAs, carrying payload miRNAs or siRNAs. Target hBERAs were purified by anion exchange FPLC method. Functions of hBERA/miRNAs were investigated in human carcinoma cells and antitumor activities were determined in orthotopic osteosarcoma xenograft spontaneous lung metastasis mouse models.

**Results:** Proper human tRNAs were identified to couple with optimal hsa-pre-miR-34a as new fully-humanized ncRNA carriers to accommodate warhead miRNAs or siRNAs. A group of 30 target hBERAs were all heterogeneously overexpressed (each accounting for >40% of total bacterial RNA), which facilitated large-scale production (8-31 mg of individual hBERAs from 1L bacterial culture). Model hBERA/miR-34a-5p and miR-124-3p were selectively processed to warhead miRNAs in human carcinoma cells to modulate target gene expression, enhance apoptosis and inhibit invasiveness. In addition, bioengineered miR-34a-5p and miR-124-3p agents both reduced orthotopic osteosarcoma xenograft tumor growth and spontaneous pulmonary metastases significantly.

**Conclusion:** This novel ncRNA bioengineering technology and resulting recombinant ncRNAs are unique additions to conventional technologies and tools for basic research and drug development.

## Introduction

MicroRNAs (miRNAs or miRs) are a superfamily of genome-derived functional small noncoding RNAs (ncRNAs) that govern posttranscriptional gene regulation to control various cellular processes 1. Some miRNAs have been identified to promote (e.g., miR-21-5p and miR-155-5p) or suppress (e.g., miR-34a-5p and miR-124-3p) human carcinoma cell proliferation and tumor progression and metastasis 2-8. Furthermore, with some exceptions, oncolytic miRNAs such as miR-34a-5p and miR-124-3p are commonly decreased or lost in human tumor tissues or cancer cells, whereas oncogenic miRNAs such as miR-21-5p are largely increased 4, 6, 9-11. Therefore, there is growing interest in developing new approaches to re-balance miRNA expression or activity towards the control of lethal cancers 12-14. Besides the utilization of miRNA mimics and antagomirs synthesized *in vitro*, efforts are also underway to identify small molecules to control the biogenesis of miRNAs from precursor miRNAs such as pri- and pre-miRs 15, 16. Further, small interfering RNAs (siRNAs) are closely related small RNAs (sRNAs) that can selectively knockdown target gene expression in cells through RNA interference (RNAi) mechanisms 17-19. While siRNAs are widely used as research tools and for therapeutic studies, three siRNA drugs namely Patisiran, Givosiran and Lumasiran have been approved very recently by the United States Food and Drug Administration for the treatment of polyneuropathy of hereditary transthyretin-mediated amyloidosis, adults with acute hepatic porphyria, and primary hyperoxaluria type 1 disorders, respectively 16, 20, 21.

**Figure 1:**
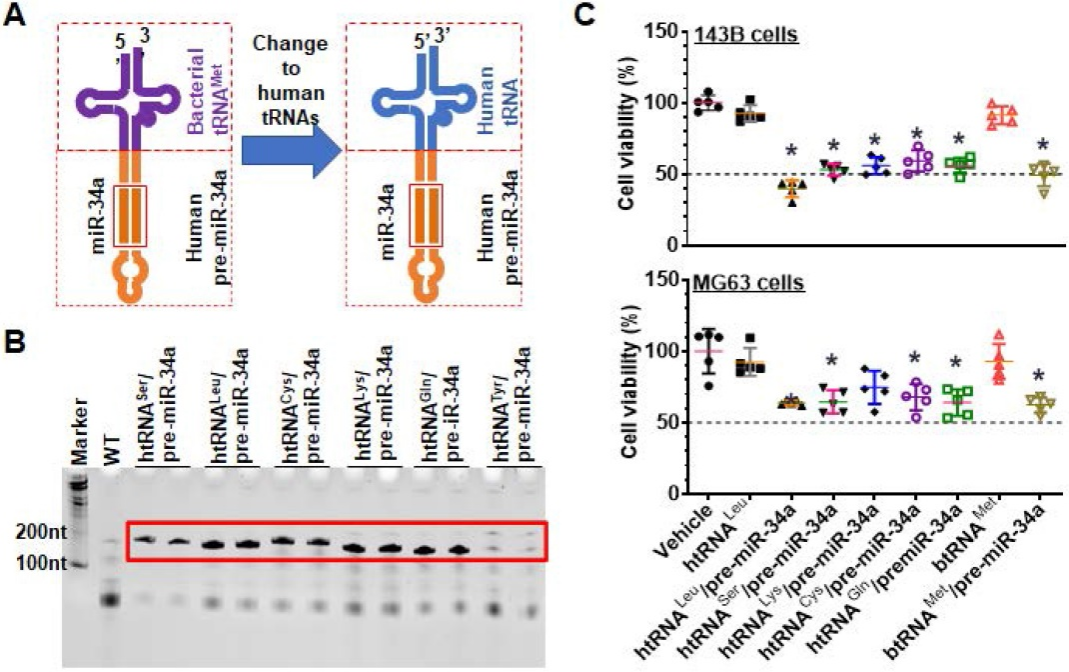
** Development of fully-humanized ncRNA carriers for *in vivo* fermentation production of biologic RNAi agents.** (**A**) Coupling a human tRNA (htRNA), instead of the bacterial tRNA (btRNA), with human pre-miR-34a offers fully-humanized ncRNA carrier. (**B**) Urea-PAGE analysis of total bacterial RNAs showed that target htRNA/hsa-pre-miR-34a molecules were highly expressed when leucyl (Leu), seryl (Ser), lysyl (Lys), cysteinyl (Cys), or glutaminyl (Gln) htRNAs were utilized. By contrast, there was minimal levels of recombinant ncRNAs when tyrosyl (Tyr) htRNA was used. (**C**) Overall, the htRNA^Leu^/pre-miR-34a exhibited relatively greater degrees of inhibition against human carcinoma 143B and MG63 cell viability. Values are mean ± SD (N = 5). *P < 0.05, as compared to vehicle, htRNA^leu^, or btRNA^Met^ controls (1-way ANOVA with Bonferroni post-tests).

Recognizing that conventional miRNA and siRNA agents utilized for research and development are primarily generated through *in vitro* chemical synthesis or enzymatic reactions 22, 23, we have taken the initiative to explore new ways to make functional ncRNA molecules through *in vivo* fermentation production. Bioengineered or recombinant ncRNA agents (BERAs) are reasoned to better recapitulate the structures and functions of natural RNAs because both are produced and folded in living cells 14, 24, similar to bioengineered or recombinant proteins that are developed and preferably used in protein research and therapy 25, 26. After the identification of a chimeric ncRNA molecule, bacterial methionyl tRNA (btRNA^Met^) fused human hsa-pre-miR-34a (btRNA^Met^/pre-miR-34a) (Fig. 1A), which can be overexpressed in bacteria 27, we established it as an optimum carrier to achieve fermentation production of recombinant ncRNAs bearing target sRNAs, including miRNAs, siRNAs and aptamers 27, 28. To make BERAs more compatible with human cells for functional and experimental therapeutic studies, our efforts were directed to developing humanized BERAs (hBERAs) with payload miRNAs or siRNAs.

In this study, we first identified that five human tRNAs (htRNAs), seryl (htRNA^Ser^), leucyl (htRNA^Leu^), lysyl (htRNA^Lys^), cysteinyl (htRNA^Cys^), glutaminyl (htRNA^Gln^), could be coupled to human pre-miR-34a (or hsa-pre-miR-34a) to achieve high-level expression of recombinant htRNA/hsa-pre-miR-34a molecules. Among them, htRNA^Leu^/pre-miR-34a and htRNA^Ser^/pre-miR-34a were utilized as humanized ncRNA carriers for the production of two collections of hBERAs consisting of warhead sRNAs (or hBERA/sRNAs), which were all highly expressed in *E. coli* (each accounting for >40% of total bacterial RNA), with a 100% success rate. Two model hBERA/sRNAs, namely htRNA^Leu^/miR-34a-5p and htRNA^Leu^/miR-124-3p, were shown to be selectively processed to miR-34a-5p and miR-124-3p, respectively, in human osteosarcoma 143B and MG63 cells to suppress corresponding cancer-related targets to achieve the enhancement of apoptosis, inhibition of cell proliferation, and suppression of invasiveness. Efficacy of biologic htRNA^Leu^/miR-34a-5p and htRNA^Leu^/miR-124-3p in the control of tumor growth was further demonstrated in a highly invasive, orthotopic osteosarcoma xenograft mouse model, as manifested by a significant reduction of tumor volume, weight, and bioluminescent signal. In addition, incidence of spontaneous pulmonary metastasis was sharply decreased in mice treated with htRNA^Leu^/miR-34a-5p and htRNA^Leu^/miR-124-3p, compared with control RNA and vehicle treatments. Our findings support the robustness of novel hBERA platform established in this study for *in vivo* fermentation production of recombinant RNAi agents, and their utilities for functional and experimental therapeutic studies.

## Materials and Methods

### Chemicals and materials

RPMI 1640 medium, phosphate-buffeted saline (PBS), 0.05% trypsin-EDTA, fetal bovine serum (FBS), opti-MEM, BCA protein assay kit, Lipofectamine 3000, and RIPA buffer were purchased from Thermo Fisher Scientific (Waltham, MA, USA). Protease inhibitor cocktail and Trizol reagent were bought from Sigma-Aldrich (St. Louis, MO, USA). Bovine Serum Albumin (BSA), in vivo-jetPEI, Thiazolyl blue tetrazolium bromide (MTT), dimethyl sulfoxide (DMSO) and crystal violet were bought from VWR (Radnor, PA, USA). Western ECL Substrate Kit, blotting-grade blocker and PVDF membrane were purchased from Bio-Rad (Hercules, CA, USA). Direct-zol RNA miniPrep kit was purchased from Zymo Research (Irvine, CA, USA). TACS Annexin V-FITC Kit was bought from Trevigen (Gaithersburg, MD, USA). Transwell invasion assay kit was bought from Corning (Corning, NY, USA). All other chemicals and organic solvents of analytical grade were purchased from Thermo Fisher Scientific, VWR or Sigma-Aldrich.

### Construction of ncRNA expression plasmids

Individual human tRNA sequences were obtained from the Genomic tRNA Database (GtRNAdb), and human miRNA sequences were extracted from miRBase. Plasmids encoding target ncRNAs (Table S1) were cloned as previously reported 28 involving two strategies. Most inserts were obtained through PCR amplification using htRNA-specific primers (Table S2) (IDT, San Diego, CA) and corresponding btRNA^Met^-based ncRNA expression plasmids^24^ as templates. A few other target inserts were directly amplified through PCR reactions with longer primers containing 16-nt complementary sequences (Table S2). The amplicons were thus cloned into pBSTNAV 29 linearized by endonucleases EcoRI-HF® and PstI-HF® (New England Biolabs, Ipswitch, MA) as previously described 28. All clones were propagated in Stellar™ Competent Cells (Takara Bio, Mountain View, CA) and confirmed by DNA sequencing (Genscript, Piscataway, NJ).

### Heterogenous expression of humanized recombinant ncRNA molecules in* E. coli*

Humanized BERAs were overexpressed in HST08 *E. coli* on small scale (15 mL) or large scale (0.5 L). To evaluate target hBERA expression, total RNAs were isolated from bacteria using the Tris-HCL-saturated phenol extraction method, quantitated with a NanoDrop 2000 spectrophotometer (Thermo Fisher Scientific), and separated by using denaturing urea (8M) polyacrylamide (8%) gel electrophoresis (PAGE). All RNA gel images were acquired with a ChemiDoc MP Imaging System (Bio-Rad, CA, USA), and intensities of RNA bands were used for the estimation of relative levels of hBERAs present in total RNAs. More accurate determination of hBERA level in total bacterial RNA was achieved by analyzing hBERA peak area over total peaks' areas from FPLC traces (see below). Two htRNA^Leu^/pre-miR-34a-carried miRNAs, miR-34a-5p and miR-124-3p that were pursued for extensive biological and therapeutic studies, as well as the control htRNALeu/Sephadex-Aptamer, were abbreviated as htRNA^Leu^/miR-34a-5p, htRNA^Leu^/miR-124-3p, and htRNA^Leu^, respectively, in the context and figures.

### Purification of fully-humanized recombinant ncRNAs

All humanized recombinant ncRNAs were purified with an ENrich^TM^ Q 10×100 column using the NGC Quest 10 Plus Chromatography fast protein liquid chromatography (FPLC) system (Bio-Rad). For the purification of chimeric hBERAs, a gradient elution was conducted at a constant flow rate of 2 mL/min, 100% Buffer A (10 mM sodium phosphate, pH = 7.0) for 5 min, and then gradually increased to 55% Buffer B (Buffer A +1 M sodium chloride) in 15 min, 55-80% Buffer B for 35 min, 100% Buffer B for 5 min, and lastly 100% Buffer A for 5 min. A different gradient elution was utilized for the purification of humanized control htRNA molecules at 2 mL/min, 100% Buffer A for 5 min, and then increased to 55% Buffer B in 15 min, 55-65% Buffer B for 35 min, 100% Buffer B for 5 min, and lastly 100% Buffer A for 5 min. FPLC traces were monitored at 260 nm using a UV/Vis detector and individual fractions were collected. Peak areas were used to evaluate the relative levels of recombinant ncRNAs within the total RNAs, which were consistent with those estimated from the above urea-PAGE analyses. Following further urea-PAGE verification, the fractions containing pure target hBERAs were pooled, precipitated with ethanol (ethanol/sample solution = 2/1, v/v), reconstituted with nuclease-free distilled deionized water, and then desalted and concentrated with Amicon ultra-0.5 ml centrifugal filter unites (30 kDa; EMD Millipore, Billerica, MA, USA).

FPLC-purified hBERAs were thus quantitated with a NanoDrop 2000 spectrophotometer. Purities of individual hBERAs were quantitatively determined by using an XBridge OST C_18_ column (2.1 × 50 mm, 2.5 μm particle size; Waters, Milford, MA) on a Shimadzu LC-20AD high-performance liquid chromatography (HPLC) system (Columbia, MD), as described 28, 30. Endotoxin levels of the isolated hBERAs were measured with the Pyrogent-5000 kinetic LAL assay (Lonza, Walkersville, MD) by following manufacturer's instructions. Briefly, a SpectraMax3 plate reader (Molecular Devices, Sunnyvale, CA) was used to measure turbidity at a 340 nm wavelength. Provided endotoxin standards were used to generate a standard curve. Bioengineered RNAs with > 97% purity (by HPLC) and endotoxin activity < 5 EU/µg RNA were used for functional and therapeutic studies.

### Human cell culture and transfection

Human osteosarcoma 143B (CRL-8303) and MG63 (CRL-1543) cell lines were purchased from American Type Culture Collection (Manassas, VA, USA). The luciferase and GFP-expressing 143B cell line (143B-Luc-GFP) was established by transduction with pCCLc-Luc-EGFP lentiviral construct (Vector Core, UC Davis Medical center, Sacramento, CA). All cells were cultured in RPMI 1640 medium supplemented with 10% FBS at 37 °C in a humidified atmosphere with 5% CO_2_ and 95% air. Cells were transfected with bioengineered/recombinant RNAs using Lipofectamine 3000 (Thermo Fisher Scientific).

### RNA isolation and reverse transcription quantitative real-time PCR (RT-qPCR)

143B and MG63 cells were treated with 10 nM of htRNA^Leu^/miR-34a-5p, htRNA^Leu^/miR-124-3p or htRNA^Leu^ for 48 h. Total RNAs were isolated using the Direct-zol RNA Miniprep Kit, and RNA concentrations were determined using a NanoDrop 2000 spectrophotometer. Reverse transcription was performed by using NxGen M-MuLV reverse transcriptase (Lucigen, Middleton, WI) with random hexamers or selective stem-loop primers for mature miRNAs as reported 28. Real-time qPCR was conducted with iTaq Universal SYBR Green Supermix (Bio-Rad) on a CFX96 Touch real-time PCR system (Bio-Rad), as described 27, 28. MiRNA levels were normalized to corresponding U6 levels with the formula 2^-ΔCt^, where ∆Ct is the difference in cycle number values between the analyte and internal standard, and compared to control treatments.

### Protein isolation and immunoblot analysis

After transfection with 10 nM htRNA^Leu^/miR34a-5p, htRNA^Leu^/miR124-3p or htRNA^Leu^ for 48 h, human OS 143B and MG63 cells were harvested for the preparation of cell lysates using RIPA buffer (Sigma Aldrich) supplemented with complete protease inhibitor cocktail (Roche Diagnostics, Mannheim, Germany). Protein concentration was determined using the BCA Protein Assay Kit (Pierce, Rockford, IL). Whole-cell lysates (40 μg proteins/lane) were separated on a 10% SDS-PAGE gel and electrotransferred onto a PVDF membrane. Membranes were incubated with individual primary antibodies against B-cell lymphoma 2 (BCL2) (1:1,000 dilution, catalog #4223S; Cell Signaling), NAD-dependent deacetylase sirtuin-1 (SIRT1) (1:100 dilution, catalog #SC-15404; Santa Cruz), hepatocyte growth factor receptor (c-MET) (1:100 dilution, catalog #SC-161; Santa Cruz), vesicle associated membrane protein 3 (VAMP3) (1:100 dilution, catalog #SC-514843; Santa Cruz), monocarboxylic acid transporter 1 (MCT1) (1:100 dilution, catalog #SC-365501; Santa Cruz), signal transducer and activator of transcription 3 (STAT3) (1:1,000 dilution, catalog #9139S; Cell Signaling), phospho-STAT3 (p-STAT3) (1:1,000 dilution, catalog #9131S; Cell Signaling), and β-actin (1:5,000 dilution, catalog #A5441; Sigma-Aldrich) overnight at 4℃, and then incubated with the secondary horseradish peroxidase-labeled anti-rabbit IgG (1:6,000 dilution, catalog #111035003; Jackson ImmunoResearch) or anti-mouse (1:3,000 dilution, catalog #7076; Cell Signaling) antibodies for 2 h at room temperature. The membranes were subsequently incubated with the Clarity Western ECL substrates (Bio-Rad) and imaged with the ChemiDoc MP Imaging System (Bio-Rad). Each experiment was repeated in triplicate. β-actin was used as a loading control.

### Cell viability assay

143B and MG63 cells were seeded in 96-well plates, and cell viability values were determined by MTT assay, as described previously 27, 28. To compare the antiproliferative activities of individual htRNA-carried miR-34a, cells were transfected with 10 nM of RNA and viability of cells treated with transfection agent/vehicle was defined as 100%. To assess the antiproliferative activities of htRNA^Leu^/miR-34a-5p and htRNA^Leu^/miR-124-3p *versus* htRNA^Leu^ and vehicle controls over time, the OD values were measured at 0, 24, 48 and 72 h post-transfection with 10 nM RNAs and utilized directly to illustrate the impact of hBERAs on cell growth.

### Apoptosis assay

Apoptotic cells were determined by flow cytometry analysis. Briefly, 143B or MG63 cells were seeded into 6-well plate at 4.5 × 10^5^ cells/well in 2 mL medium, and treated with 10 nM htRNA^Leu^/miR34a-5p, htRNA^Leu^/miR124-3p or htRNA^Leu^ for 48 h. Cells were harvested and washed twice with cold PBS and then stained with Annexin V-FITC and propidium iodide solution (Trevigen Inc., Gaithersburg, MD) according to the manufacturer's instructions. Samples were analyzed on a FACS Canto flow cytometer (BD Biosciences, San Jose, CA), and all data were processed by Flowjo (Ashland, OR).

### Cell invasion assay

Cells were seeded in 6-well plates and transfected with 10 nM of htRNA^Leu^/miR-34a-5p, htRNA^Leu^/miR-124-3p or htRNA^Leu^. After 48 h, survived cells were harvested and seeded onto the upper inserts of the 24-well Corning BioCoat Matrigel Invasion Chambers (Corning, Bedford, MA) at 6 × 10^4^ cells/well with 500 μL serum-free RPMI 1640 medium where the lower chamber was supplied with 750 μL of serum-containing culture medium (10% FBS). 20 h later, the invaded cells were fixed with 500 μL of 10% formaldehyde, stained with 500 μL 0.1% crystal violet, and imaged with an Olympus IX2-UCB microscope. The number of invaded cells was acquired by counting five fields per insert (100 × magnification), and then compared between different treatments as we described recently 31.

### Orthotopic xenograft spontaneous lung metastasis mouse models and RNA therapy study

All animal procedures were approved by the Institutional Animal Care and Use Committee at UC Davis and all studies were performed according to Guide for the Care and Use of Laboratory Animals issued by the National Institutes of Health. 5-week-old female mice (NOD.CB17-Prkdc^scid^/J) were bought from The Jackson Laboratory (Bar Harbor, ME) and housed in a pathogen-free facility at UC Davis. After the mice were acclimated for 1 week, orthotopic osteosarcoma xenograft spontaneous lung metastasis mouse model was established as we described recently 32. Briefly, human OS 143B-GFP-Luc cells in logarithmic growth were harvested and resuspended in PBS to a final density of 2 × 10^7^ cells/mL. Forty μL of the cell suspension (8 × 10^5^ cells) were injected into the right tibial medullary cavity of anesthetized mice. Tumor development was monitored through live animal bioluminescence imaging as well as measuring with a caliper. Particularly, anesthetized mouse was injected intraperitoneally with D-luciferin (150 mg/kg) 5 min before imaging, and images were acquired with a ChemiDoc MP Imaging System (Bio-Rad). Following caliper measurement, tumor volume was calculated using the formula, (length + width) × length × width × 0.2618.

When bioluminescence signal confirmed the outgrowth of orthotopic OS xenograft which occurred at 9 days post-inoculation, 40 mice showing similar degrees of bioluminescence intensity were enrolled and randomly divided into 4 groups (N = 10/group). On day 10 post-inoculation, mice were administered intravenously with in vivo-jetPEI (Polyplus-transfection Inc., New York, NY) formulated htRNA^Leu^/miR-34a-5p, htRNA^Leu^/miR-124-3p, control htRNA^Leu^ at a dose of 30 μg/mouse, or control vehicle every other day. On day 28, all mice were euthanized and the tibial xenograft tumors were carefully removed and weighed. Furthermore, individual lung tissues were dissected immediately, subjected to *ex vivo* bioluminescence imaging, and then fixed in 10% formalin and subjected to hematoxylin and eosin (H&E) staining for histological evaluation in the Clinical Immunohistochemistry Laboratory at Roswell Park Cancer Institute (Buffalo, NY, USA).

### Statistical analysis

Values are mean ± S.D., and all data were analyzed with one-way or two-way ANOVA or comparison of proportions (GraphPad Prism). When* P* value was less than 0.05 (*P* < 0.05), the difference was considered as statistically significant.

## Results

### Identification of humanized ncRNA carriers for high-level heterogeneous expression of recombinant ncRNAs

To develop humanized ncRNA carriers for the production of recombinant ncRNAs (Figure 1A), we first identified and utilized several htRNAs that are highly expressed in healthy human cells 33, namely htRNA^Ser^, htRNA^Leu^, htRNA^Lys^, htRNA^Cys^, htRNA^Tyr^, and htRNA^Gln^, to replace the bacterial btRNA^met^ toward humanization. Target htRNA/hsa-pre-miR-34a expression plasmids were cloned and confirmed, and then transformed into HST08 *E. coli* competent cells to assess at what levels recombinant ncRNAs could be expressed. Urea PAGE analyses of the total bacterial RNAs revealed that, except htRNA^Tyr^/pre-miR-34a, all other target ncRNAs including htRNA^Leu^/pre-miR-34a, htRNA^Ser^/pre-miR-34a, htRNA^Lys^/pre-miR-34a, htRNA^Cys^/pre-miR-34a, and htRNA^Gln^/pre-miR-34a were efficiently accumulated in *E. coli* to a surprisingly high level, i.e., accounting for more than 40% of total bacterial RNA (Figure 1B), indicating successful heterogeneous overexpression of fully-humanized tRNA/pre-miRNA molecules.

To choose specific htRNA/hsa-pre-miRNA as carrier for further studies, the inhibitory activities of five htRNA-carried hsa-pre-miR-34a molecules against human carcinoma 143B and MG63 cell viability were determined. All recombinant miR-34a agents showed significant inhibition in both cell lines, as compared to vehicle or tRNA controls (Figure 1C). Overall, htRNA^Leu^ loaded pre-miR-34a showed a relatively greater degree of activity against both cell lines among these recombinant miR-34 agents. Therefore, htRNA^Leu^/hsa-pre-miR-34a was selected as a carrier for further studies while htRNA^Ser^/hsa-pre-miR-34a was also utilized for the production of hBERAs since both htRNA^Leu^ and htRNA^Ser^ are highly abundant in healthy human cells 33, 34.

### Application of new ncRNA carriers to *in vivo* fermentation production of recombinant RNAi agents

**Figure 2:**
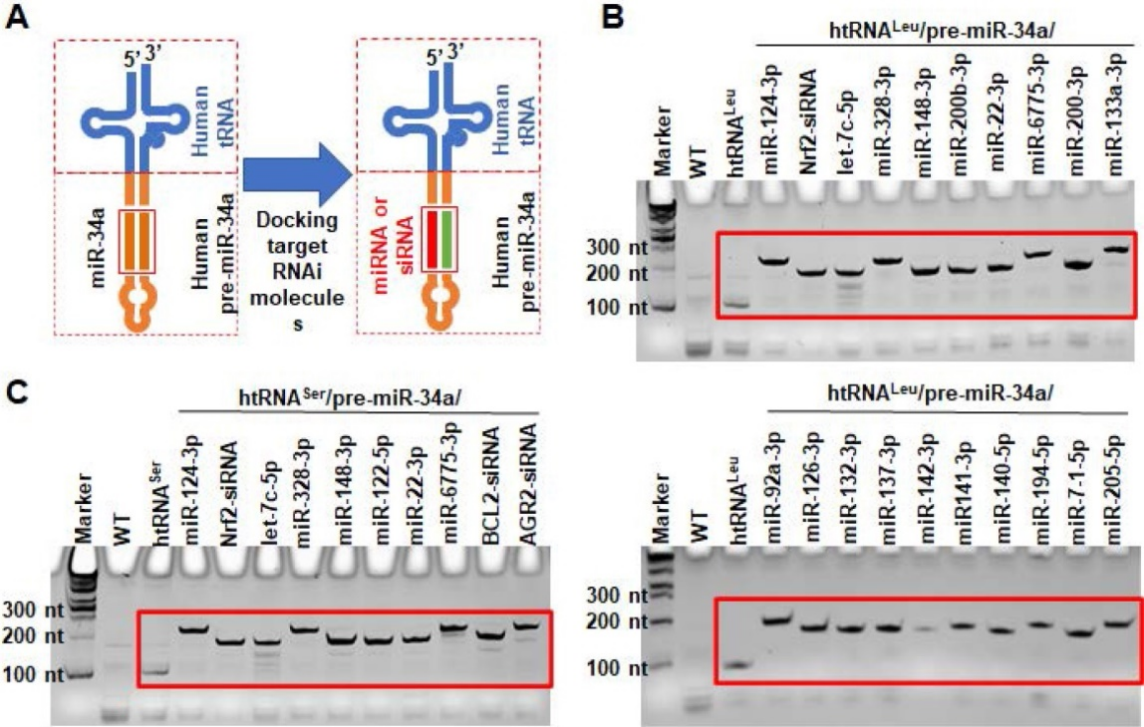
** Utilization of fully-humanized ncRNA carriers to achieve *in vivo* fermentation production of biologic RNAi agents.** (**A**) Substituting miR-34a duplexes within the htRNA/hsa-pre-miR-34a carrier with target miRNA or siRNA offers humanized RNAi agents or hBERAs. (**B** and **C**) Two sets of target hBERAs were all highly expressed in bacteria, using either htRNA^Leu^/hsa-pre-miR-34a or htRNA^Ser^/hsa-pre-miR-34a as a carrier. Total RNA from wild type *E. coli* was used for comparison.

To produce target biologic RNAi agents or hBERAs, the miR-34a duplexes within humanized tRNA/pre-miRNA carrier were replaced with a warhead miRNA or siRNA and complementary sequences (Figure 2A). A total of 30 hBERAs (Table S1) were thus designed by using htRNA^Leu^/pre-miR-34a and htRNA^Ser^/pre-miR-34a carriers, and corresponding coding sequences were cloned into the pBSTNAV vector 28, 29 using specific primers (Table S2). After confirmation by DNA sequencing, bacteria were transformed with hBERA-expressing plasmids and total RNAs were analyzed by urea-PAGE to evaluate the expression of individual hBERAs. Compared to wild-type bacterial total RNA as well as control htRNA^Leu^ and htRNA^Ser^, appearance of new strong RNA bands at expected sizes (~200 nt) demonstrated overexpression of individual target hBERAs, with a 100% success rate (Figure 2C-D). In addition, each hBERA was revealed to account for 40-80% of total bacterial RNA, supporting the utility of particular htRNA/hsa-pre-miR-34a carriers to achieve high-level expression of recombinant RNAi agents.

### Purification of hBERAs to a high degree of homogeneity

**Figure 3:**
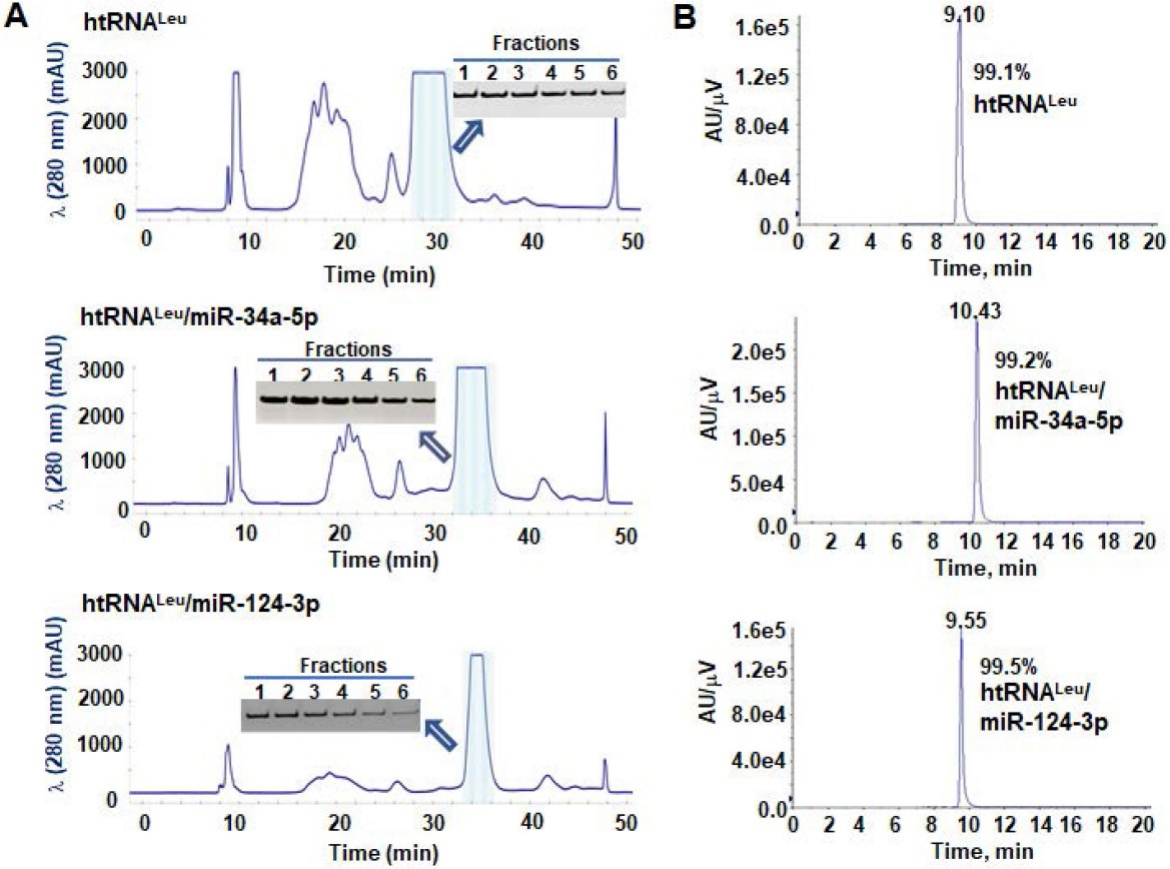
** FPLC purification of fermentation-produced miRNA agents and HPLC determination of recombinant ncRNAs purities.** (**A**) FPLC-UV traces during the purification of htRNA^Leu^/miR-34a-5p, htRNA^Leu^/miR-124-3p, and control htRNA^Leu^. The insert shows urea-PAGE analysis of corresponding target RNA fractions #1 to 6. (**B**) The purities of final htRNA^Leu^/miR-34a-5p, htRNA^Leu^/miR-124-3p, and control htRNA^Leu^ molecules were determined by HPLC analyses, which are all over 99% pure.

To isolate hBERAs for functional and experimental therapeutic studies, two anion exchange FPLC methods were established for the purification of target hBERAs and control tRNAs using an ENrich-Q 10 × 100 column (Figure 3A). Purities of FPLC-isolated ncRNA products were quantitatively determined by HPLC method (Figure 3B). The results showed that the majority of FPLC-purified hBERAs are highly homogenous (> 97%), with low endotoxin activity (< 5 EU/µg RNA) (Table 1). This new hBERA bioengineering platform generally produced 20-40 mg total RNAs from 1 L bacterial culture, which offered 8-31 mg pure hBERAs after purification (equivalent to 20-75% overall yield). Interestingly, while both htRNA^Leu^/pre-miR-34a and htRNA^Ser^/pre-miR-34a carriers offered comparable high-level expression of target hBERAs (Figure 2C-D), the overall yields of htRNA^Leu^/pre-miR-34a-carried molecules (18.6 ± 6.7 mg RNA/L culture) were significantly (P < 0.01, Student's t-test) higher than htRNA^Ser^/pre-miR-34a series (11.8 ± 3.2 mg RNA/L culture) (Table 1), which was associated with a lower yield of total RNAs isolated from bacteria expressing the htRNA^Ser^ serie of hBERAs. Therefore, htRNA^Leu^/pre-miR-34a appears superior to htRNA^Ser^/pre-miR-34a to offer higher-yield production of target hBERAs.

### Payload miR-34a-5p and miR-124-3p are selectively released from hBERAs in human carcinoma cells to control target gene expression and cell proliferation

**Figure 4:**
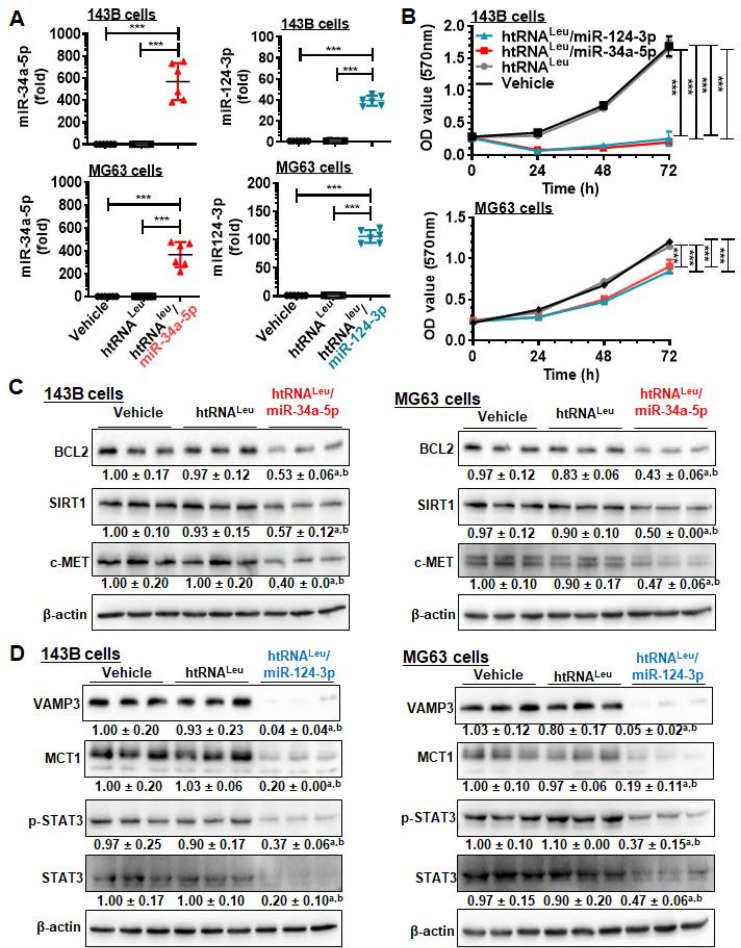
** Bioengineered miR-34a-5p and miR-124-3p molecules are effective to modulate target gene expression and control human carcinoma cell proliferation.** (**A**) Levels of miR-34a-5p and miR-124-3p were sharply higher in cells treated with htRNA^Leu^/miR-34a-5p and htRNA^Leu^/miR-124-3p, respectively, as compared with vehicle or htRNA^Leu^ control. MiRNA levels were determined by selective stem-loop RT-qPCR assays. Values are the mean ± SD (N = 6/group). ***P < 0.001, 1-way ANOVA with Bonferroni post-tests. (**B**) Humanized miR-34a and -124 prodrugs greatly reduced the proliferation of carcinoma cells. Values are the mean ± SD (N = 5/group). ***P < 0.001, 2-way ANOVA with Bonferroni post-tests). (**C**) and (**D**) Protein levels of miR-34a (BCL2, SIRT1, and c-MET) and miR-124 (VAMP3, MCT1, and STAT3) targets were significantly reduced in cells treated with miR-34a-5p and miR-124-3p prodrugs, respectively, compared to control treatments. Band intensity was normalized to corresponding β-actin level. *^a^P* < 0.05 compared to vehicle treatment, and *^b^P* < 0.05 compared to htRNA^Leu^; 1-way ANOVA with Bonferroni post-tests.

We then sought to use htRNA^Leu^/miR-34a-5p (or hBERA/miR-34a-5p) and htRNA^Leu^/miR-124-3p (hBERA/miR-124-3p) as model hBERAs for functional and therapeutic studies since miR-34a-5p 4, 35-38 and miR-124-3p 7, 8 are well-established oncolytic miRNAs. Our data showed that htRNA^Leu^/miR-34a-5p and htRNA^Leu^/miR-124-3p were precisely processed to high levels of mature miR-34a-5p and miR-124-3p, respectively, in human carcinoma cells (Figure 4A), leading to 90-95% inhibition of 143B cell viability and 25-30% suppression of MG63 cell viability (Figure 4B). This was associated with significant reduction of protein levels of BCL2, SIRT1 and c-MET (approximately 40-60%) by hBERA/miR-34a-5p (Figure 4C), and VAMP3, MCT1, p-STAT3 and STAT3 (about >50%) by hBERA/miR-124-3p (Figure 4D), compared to vehicle and htRNA^Leu^ control treatments. These results demonstrated that hBERAs were precisely processed to target miRNAs in human carcinoma cells to modulate target gene expression and control cancer cell proliferation.

### Recombinant miR-34a-5p and miR-124-3p molecules enhance apoptotic cell death and suppress invasion capacity

**Figure 5:**
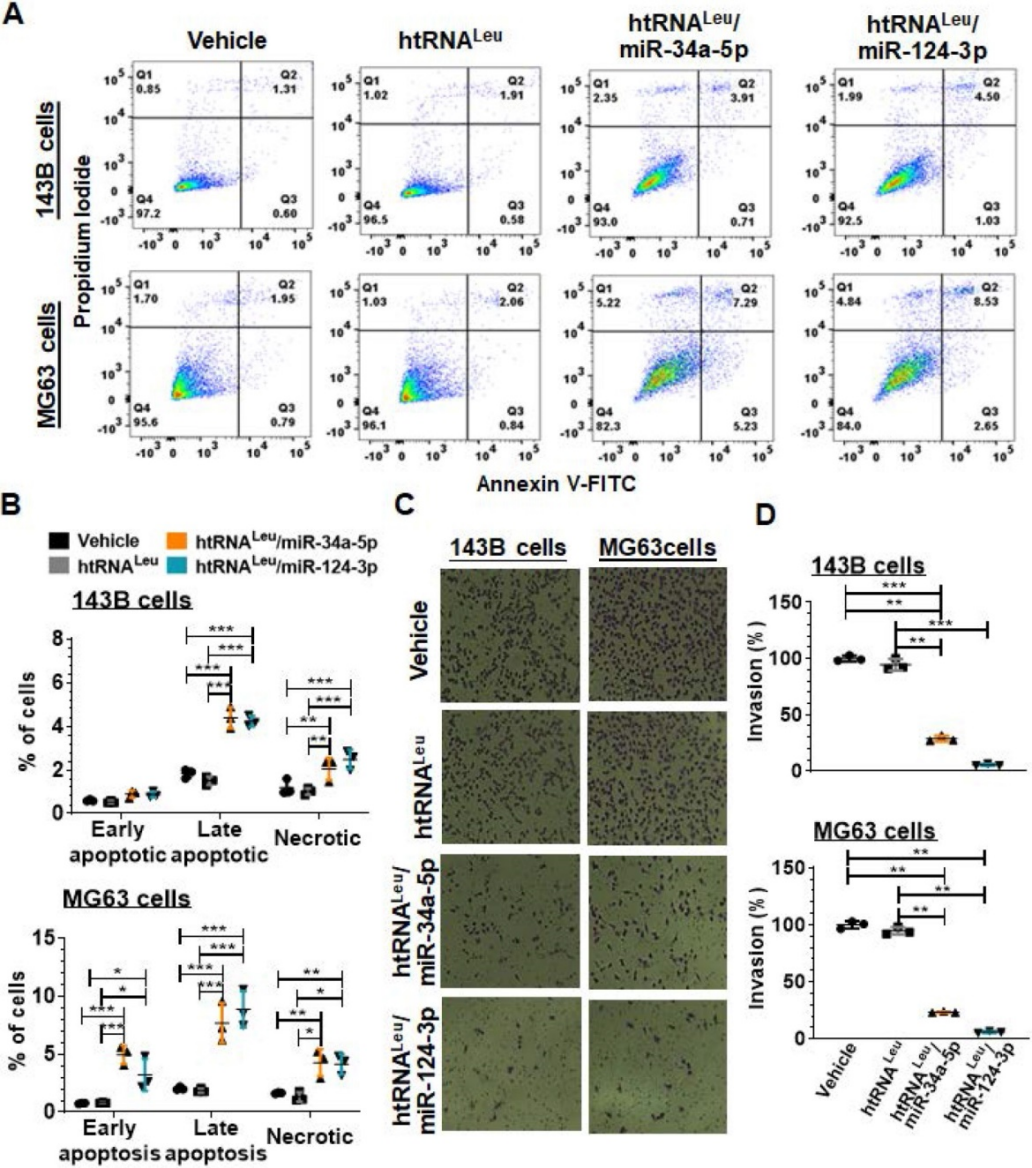
**Fully-humanized biologic miR-34a-5p and miR-124-3p agents enhance apoptotic cell death and inhibit invasion capacity of human carcinoma cells.** (**A**) Comparison of flow cytometry histograms of Annexin V/PI-stained 143B and MG63 cells at 48 h post-transfection with 10 nM htRNA^Leu^/miR34a-5p, htRNA^Leu^/miR124-3p, control htRNA^Leu^ or vehicle. (**B**) The percentage of late apoptotic cells was increased sharply by bioengineered miR-34a or miR-124 agents. So was the fraction of necrotic cells. Furthermore, htRNA^Leu^/miR-34a-5p and miR-124-3p significantly reduced the invasion abilities of 143B and MG63 cells, as indicated by cell images (**C**; 100 × magnification) and quantitative comparison of the number of invaded cells (**D**). Values are mean ± SD (N = 3 per group). *P < 0.05, **P < 0.01, and ***P < 0.001; 2- or 1-way ANOVA with Bonferroni post-tests.

We further examined the apoptotic profiles of hBERA-treated carcinoma cells (Figure 5A) by using flow cytometry, and our data showed that the percentage of late apoptotic cells at 48 h post-transfection with hBERA/miR-34a-5p or miR-124-3p was increased distinctly by 2- to 4-fold, compared to corresponding 143B or MG63 cells treated with vehicle and control htRNA^Leu^ (Figure 5B). Meanwhile, both hBERA/miR-34a-5p and miR-124-3p induced cell necrosis to 1- to 1.5-fold higher levels than vehicle and htRNA^Leu^ control treatments.

Since tumor cells usually migrate and invade through the extracellular matrix to metastasize, we further employed cell invasion assays to investigate how recombinant miR-34a-5p and miR-124-3p would affect the invasion capacity of 143B and MG63 cells. Our results showed that htRNA^Leu^/miR-34a-5p reduced the invasion ability of 143B and MG63 by 70% and 80%, respectively, as compared to vehicle and htRNA^Leu^ control treatments (Figure 5C-D). Interestingly, htRNA^Leu^/miR-124-3p showed a slightly greater inhibition (around 90%) of invasive ability of both 143B and MG63 cells than miR-34a-5p (about 75%). Together, these results exemplified the actions of humanized recombinant miRNAs in the control of cancer cell properties.

### Humanized miR-34a-5p and miR-124-3p prodrugs inhibit orthotopic osteosarcoma xenograft tumor growth and spontaneous lung metastasis in mouse models

**Figure 6:**
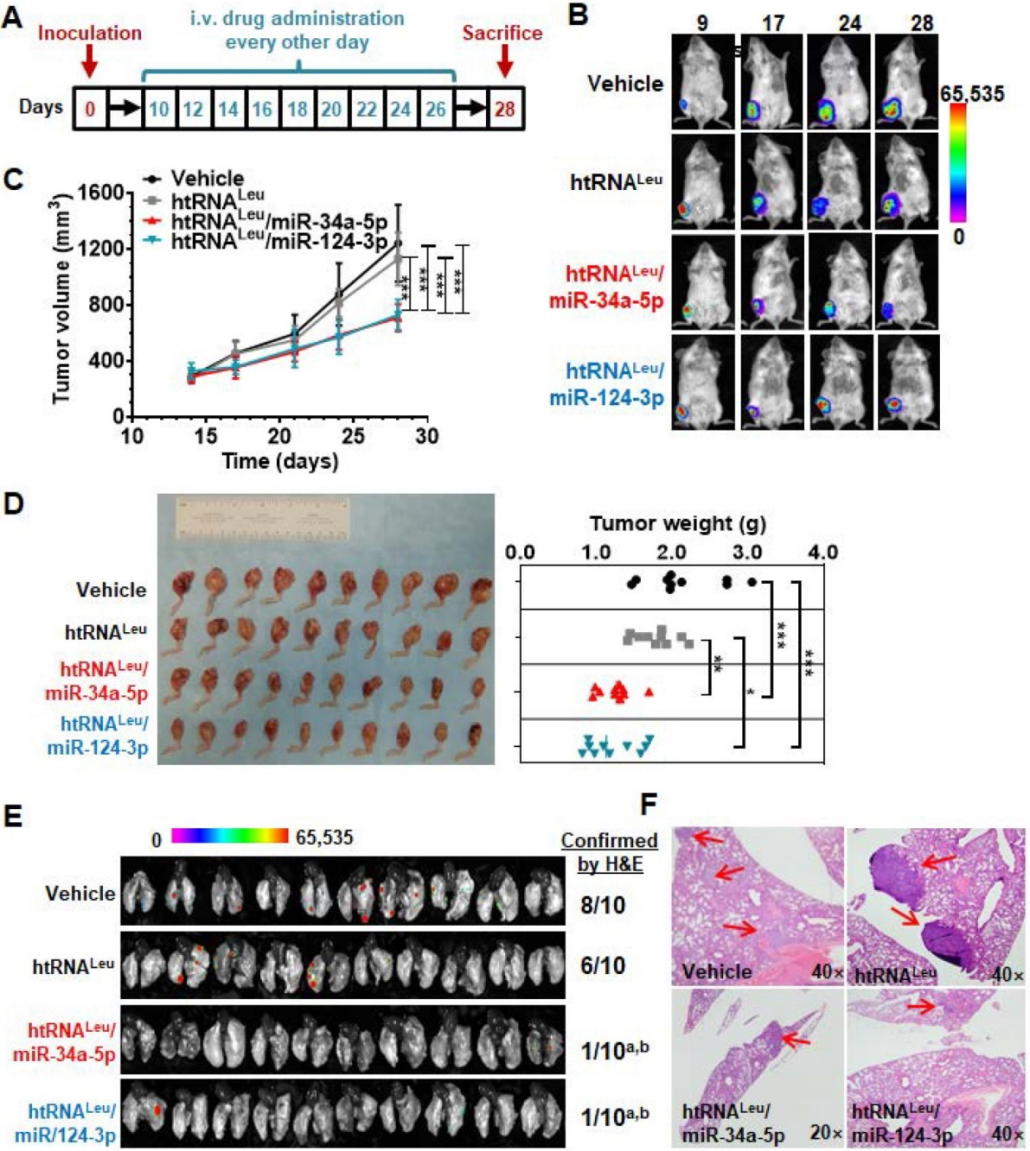
**Humanized miR-34a-5p and miR-124-3p prodrugs largely reduce orthotopic osteosarcoma xenograft tumor growth and spontaneous pulmonary metastasis in mouse models**. (**A**) Timeline of the therapy study. (**B** and **C**) Biologic miR-34a or -124 prodrug treatment largely inhibited the growth of orthotopic osteosarcoma xenograft tumors in mice, compared with vehicle or htRNA control, as examined by live animal bioluminescent imaging and caliper measurement, respectively. Values are mean ± SD (N = 10 per group; ***P < 0.001, compared to either htRNA or vehicle treatment; 2-way ANOVA). (**D**) Visual comparison of dissected orthotopic tumor tissues from mice with different treatments, as well as weights (*P <0.05, *P < 0.01, and ***P < 0.001, 1-way ANOVA with Bonferroni post-tests). (**E**) Bioluminescent imaging of *ex vivo* whole lung tissues revealed a much lower incidence of pulmonary metastasis in miR-34a and -124 treated mice, which was further confirmed by H&E staining. *^a^P* < 0.01, compared to vehicle control; *^b^P* < 0.05 compared to htRNA^leu^ treatment (Comparison of proportions). (**F**) Representative H&E staining. 40× or 20× magnification; metastasis focuses indicated by red arrows.

The 143B cells are comprised of a *p53* mutant commonly found in human malignant osteosarcoma, serving as proper models for investigating sarcoma biology and new therapeutics, whereas MG63 cells were not tumorigenic *in vivo* 39. We thus established an aggressive orthotopic osteosarcoma 143B xenograft tumor and spontaneous lung metastases mouse model, as reported 31, 32, 39, to define the antitumor efficacy of bioengineered miR-34a-5p and miR-124-3p prodrugs. A cohort of 40 SCID mice were inoculated intratibially with 143B-Luc-GFP cells to outgrow orthotopic xenograft tumors that were monitored weekly by bioluminescence imaging and using calipers. Tumor-bearing mice were thus randomly divided into four groups to receive drug (htRNA^Leu^/miR-34a-5p and htRNA^Leu^/miR-124-3p) or control (htRNA^Leu^ and vehicle) treatments (Figure 6A). Our results revealed that, compared with vehicle and htRNA^Leu^ control treatments, systemic administration of htRNA^Leu^/miR-34a-5p or htRNA^Leu^/miR-124-3p markedly suppressed xenograft tumor growth in mice, as manifested by much weaker bioluminescence signals (Figure 6B) and smaller caliper-measured tumor sizes (Figure 6C) over the course of treatment. The effectiveness of bioengineered miR-34a-5p and miR-124-3p prodrugs in the control of orthotopic xenograft tumor progression was further demonstrated by visual inspection of the dissected xenograft tumors and quantitative measurement of tumor weights (Figure 6C) from different treatment groups at the end of the study. Meanwhile, all treatments were well tolerated, given the findings that all mice did not display any signs of severe stress (e.g., hunched posture or labored movement, etc.) but retained steady body weights throughout the duration of the study that do not differ between any treatments (Figure S1).

Lastly, we dissected lung tissues from individual mice to assess the effectiveness of htRNA^Leu^/miR-34a-5p and htRNA^Leu^/miR-124-3p in the control of spontaneous lung metastases, a common phenomena and the major cause of death. As determined by *ex vivo* bioluminescence imaging of whole lung tissues (Figure 6E), 6-8 out of 10 mice receiving vehicle or control RNA treatment clearly showed lung metastases, whereas only 1-2 out of 10 mice receiving biologic miRNA prodrug therapy exhibited obvious lung metastases. Further histopathological study confirmed the presence of lung metastases of individual animals (Figure 6F), except that one lung tissue from the miR-124-3p treatment group displayed scrawny bioluminescence signal whereas it was not confirmed by histopathological examination. These results demonstrate that humanized recombinant miR-34a-5p and miR-124-3p prodrugs were effective to protect mice from distant pulmonary metastasis, besides the reduction of outgrowth of orthotopic osteosarcoma.

## Discussion

With the discovery of functional ncRNAs derived from the genome in cells, there are growing interests in understanding their roles in human diseases including lethal cancer and thus developing new RNA-based therapies 13, 16, 20, 40. For instance, oncolytic miRNAs lost or downregulated in carcinoma cells may be restored for the control of tumor progression and metastasis. This “miRNA replacement therapy” strategy is of particular interest, compared to “antagonism” of oncogenic miRNAs overexpressed in tumor tissues because miRNAs are endogenous components and miRNAs reintroduced into the body and cells may be well tolerable 12. The premise of miRNA replacement therapy strategy has been demonstrated by many preclinical studies, and some oncolytic miRNAs have entered into clinical investigations 41, 42. However, current miRNA research and drug development are dominated by the use of chemo-engineered miRNA mimics made *in vitro*, which is in sharp contrast to protein research and drug development that has been proven successful by utilizing bioengineered or recombinant proteins made and folded in living cells rather than synthetic proteins or polypeptides made *in vitro* 14, 24. Therefore, new approaches are highly demanded to achieve *in vivo* production of true biologic RNA agents, as this novel hBERA platform technology we report herein, for critical research on RNA functions and therapeutic potential. These bioengineered RNA molecules should better recapitulate the structural, physicochemical and biological properties of natural RNAs produced and folded in living cells.

The specific htRNA/hsa-pre-miR-34a carriers identified in this study was able to accommodate a wide variety of payload miRNAs (and siRNAs and other sRNAs) to achieve consistent high-yield and large-scale *in vivo* fermentation production of hBERA/RNAi agents. This is an important improvement from hybrid btRNA/hsa-pre-miRNA carrier we developed very recently 28 since all RNA sequences within the hBERA carrier are normal constituents of human cells. Moreover, all target hBERAs were successfully overexpressed in *E. coli*, offering a 100% success rate which should be challenged by future studies on more constructs. In addition, an improved expression of hBERAs (>40% of total bacterial RNA) also facilitated the purification and increased overall yield that led to 8-31 mg of highly-homogeneous ready-to-use hBERAs from one liter of bacterial fermentation, superior to previous btRNA/hsa-pre-miRNA carrier 28. It is also noteworthy that the htRNA^Leu^/hsa-pre-miR-34a carrier was better than htRNA^Ser^/hsa-pre-miR-34a to achieve higher overal yields of pure hBERAs, although the releative expression levels of target hBERAs to total bacterial RNAs were comparable by using the two carriers. This difference is likely attributable to the lower levels of total RNAs from htRNA^Ser^/hsa-pre-miR-34a-transformed bacteria, suggesting that htRNA^Ser^/hsa-pre-miR-34a might be relatively more toxic to the host bacteria and indicating the advantages of using htRNA^Leu^/hsa-pre-miR-34a carrier for *in vivo* fermentation production of biolgoic RNAi agents.

The effectiveness of biologic hBERA/miRNAs in the suppression of tumor progression and pulmonary metastasis was further demonstrated in animal models in this study. Two well-established oncolytic miRNAs commonly downregulated in human carcinoma cells, miR-34a-5p 4, 35-37 and miR-124-3p 7, 8, were chosen as model miRNAs. Considering that osteosarcoma is the most common primary malignant bone tumor among adolescents and young adults, and its high rate of pulmonary metastasis is the main cause of death 43-45, the orthotropic osteosarcoma xenograft and spontaneous metastasis mouse model 31, 32 was utilized in the present study. Indeed, tumor suppressive miR-34a-5p and miR-124-3p have been found to be downregulated in osteosarcoma patient specimens and cells 46. Our studies revealed that payload miR-34a-5p and miR-124-3p were both able to significantly suppress xenograft tumor growth in mice, compared with control RNA or vehicle treatments. Moreover, our results showed that hBERA/miR-34a-5p and miR-124-3p were equally effective in reducing lung metastasis, as determined by bioluminescent imaging and histopathological studies. These findings support the therapeutic potential of novel hBERA agents obtained through *in vivo* fermentation production platform technology while more extensive investigations such as impact on overall survival and effects in other animal models are warranted.

The antitumor activities of hBERA/miRNAs are also consistent with their mechanistic actions demonstrated in osteosarcoma cells *in vitro*. Payload miR-34a-5p and miR-124-3p were precisely released from respective hBERAs in osteosarcoma cells, and subsequently inhibited cell viability over time. The latter was associated with significant enhancement of apoptosis. Furthermore, biologic miR-124-3p exhibited stronger antiinvasive effects than miR-34a-5p against both 143B and MG64 cells. The functions of hBERAs in the control of apoptosis, proliferation and invasiveness were contributed at least partially to the downregulation of a number of cancer-related genes such as BCL2, SIRT1 and c-MET by bioengineered miR-34a-5p, and MCT1, STAT3, and VAMP3 by recombinant htRNA^Leu^/miR-124-3p, in line with previous findings on the control of target gene expression in cancer cells by genome-derived miR-34a-5p 35, 36 and miR-124-3p 7, 8. Our studies support that hBERA/miRNAs produced through microbial fermentation are biologically active to regulate target gene expression and control cancer cellular processes, supporting the utilities of bioengineered RNAs for functional studies. In addition, recombinant pre-miRNA molecules may be directly utilized for the identification of small-molecule binders 15 which awaits further investigation.

This hBERA platform seems limited to the accommodation of payload sRNAs around 18-25 nt in length during microbial fermentation production, while the release of warhead sRNAs in human cells to exert RNAi actions may or may not be dependent on Dicer 28. Albeit that the htRNA/hsa-pre-miRNA carriers identified in the present study have shown some advantages over previous btRNA/pre-miRNA carriers in producing target RNAi molecules, further studies are warranted to critically compare the efficiency of using fully-humanized and hybrid ncRNA carriers in delivering payload sRNAs into target cells, as well as safety profiles. In addition, our previous studies have revealed that the btRNA segment of recombinant ncRNAs produced in bacteria is indeed comprised of a number of posttranscriptional modifications, among them some are common and well conserved across species while several occur mainly on bacterial tRNAs 30. On the other hand, the hsa-pre-miR-34a section within BERAs is totally free of any posttranscriptional modification while exhibiting greater activities than chemo-engineered counterparts in the inhibition of cancer cell viability and regulation of target gene expression 30. Future studies shall critically delineate whether htRNA/hsa-pre-miR-34a-based molecules and respective htRNAs expressed and purified from bacterial culture are comprised of the same posttranscriptional modifications well-conserved between bacterial and human/eukaryotic tRNAs or any bacterial modification(s), and if so, whether that bacterial modification(s) affects the overall functions and safety of payload miRNAs. A head-to-head comparison of the efficacy and selectivity of bio- and chemo-engineered miRNAs in the regulation of target gene expression as well as safety profiles will improve the understanding of utilities of biologic RNA agents produced *in vivo*.

## Conclusions

This novel ncRNA bioengineering platform technology using specific htRNA/hsa-pre-miR-34a carriers identified in this work offers a consistent high-yield, large-scale and *in-vivo* fermentation production of humanized RNA molecules consisting of payload sRNAs of interest. Recombinant ncRNAs are readily purified to high degree of homogeneity by using anion exchange FPLC methods, offering tens milligrams of ready-to-use hBERAs from one liter of bacterial culture. Functional studies of two model hBERA/miRNAs, miR-34a-5p and miR-124-3p, indicate that hBERA/miRNAs are selectively processed to mature miRNAs to modulate target gene expression and cellular processes in human carcinoma cells *in vitro*. In addition, hBERA/miR-34a-5p and miR-124-3p were effective to reduce tumor growth and lung metastasis in orthotopic osteosarcoma xenograft and spontaneous metastasis mouse models* in vivo*. This robust and versatile hBERA platform offers a novel means beyond current technologies for the production of biologic RNAi agents for functional and experimental therapeutic studies.

## Supplementary Material

Supplementary figure and tables.

## Figures and Tables

**Table 1:** Yields and purities of bioengineered miRNA and siRNA agents produced on a large scale using humanized tRNA/pre-miR-34a carriers, and isolated by anion exchange FPLC methods

Bioengineered RNAi agents	Yield (mg RNA/L fermentation)	Purity (%, by HPLC)	Endotoxin activity (EU/µg RNA)
htRNA^Lys^/pre-miR-34a-5p	19.2	99.3	0.2
htRNA^Cys^/pre-miR-34a-5p	10.5	98.6	0.75
htRNA^Gln^/pre-miR-34a-5p	14.0	97.6	0.78
htRNA^Leu^/pre-miR-34a-5p	15.5	99.6	2.52
htRNA^Ser^/pre-miR-34a-5p	11.5	99.2	4.4
htRNA^Leu^	15.0	99.5	3.0
htRNA^Leu^/miR-124-3p	20.0	99.5	4.0
htRNA^Leu^/let-7c-5p	18.0	94.9	1.0
htRNA^Leu^/NRF2-siRNA	17.6	99.3	0.26
htRNA^Leu^/miR-200b-3p	31.2	98.7	0.18
htRNA^Leu^/miR-148-3p	15.3	98.7	0.05
htRNA^Leu^/miR-22-3p	17.6	97.6	0.08
htRNA^Leu^/miR-6775-3p	18.5	98.7	3.2
htRNA^Leu^/miR-133a-3p	26.6	98.2	3.0
htRNA^Leu^/miR-122-5p	26.6	97.6	3.04
htRNA^Leu^/miR-328-3p	19.0	97.8	0.35
htRNA^Leu^/ miR-7-1-5p	10.5	99.5	5.88
htRNA^Leu^/ miR-92a-3p	12.9	99.8	7.27
htRNA^Leu^/ miR-126-3p	24.3	99.7	1.51
htRNA^Leu^/ miR-132-3p	16.4	99.8	1.41
htRNA^Leu^/ miR-137-3p	27.5	99.9	0.66
htRNA^Leu^/ miR-140-5p	6.1	99.9	7.92
htRNA^Leu^/ miR-141-3p	14.7	99.9	1.55
htRNA^Leu^/ miR-142-3p	5.3	88.6	3.33
htRNA^Leu^/ miR-194-3p	20.0	99.4	3.22
htRNA^Leu^/ miR-205-5p	24.4	99.8	1.89
htRNA^Ser^	6.5	99.6	4.27
htRNA^Ser^/BCL2-siRNA	9.6	91.9	0.05
htRNA^Ser^/NRF2-siRNA	14.0	99.2	0.1
htRNA^Ser^/AGR2-siRNA	12.0	98.8	1.5
htRNA^Ser^/miR-124-3p	14.5	98.9	1.9
htRNA^Ser^/let-7c-5p	9.5	96.7	0.8
htRNA^Ser^/miR-328-3p	17.0	98.5	5.0
htRNA^Ser^/miR-200b-3p	8.4	98.8	1.3
htRNA^Ser^/miR-22-3p	8.5	99.1	1.8
htRNA^Ser^/miR-148-3p	16.2	98.9	0.1
htRNA^Ser^/miR-6775-3p	8.66	99.2	0.5

## Abbreviations

BCL2: B-cell lymphoma 2
btRNA: bacterial transfer RNA
c-MET: hepatocyte growth factor receptor
*E. coli*: *Escherichia coli*
FPLC: fast protein liquid chromatograph
hBERA: humanized bioengineered RNA agent
htRNA: human transfer RNA
htRNA^Ser^: human seryl tRNA
htRNA^Leu^: human leucyl tRNA
htRNA^Lys^: human lysyl tRNA
htRNA^Cys^: human cysteinyl tRNA
htRNA^Gln^: human glutaminyl tRNA
MCT1: monocarboxylic acid transporter 1
miR or miRNA: microRNA
ncRNA: noncoding RNA
p-STAT3: phospho-STAT3
RNAi: RNA interference
sRNA: small RNA
siRNA: small interfering RNA
SIRT1: NAD-dependent deacetylase sirtuin-1
tRNA: transfer RNA
STAT3: signal transducer and activator of transcription 3
VAMP3: vesicle associated membrane protein 3

## References

[1] Ambros V (2004). The functions of animal microRNAs. Nature.

[2] Chan JA, Krichevsky AM, Kosik KS (2005). MicroRNA-21 is an antiapoptotic factor in human glioblastoma cells. Cancer Res.

[3] Gironella M, Seux M, Xie MJ, Cano C, Tomasini R, Gommeaux J (2007). Tumor protein 53-induced nuclear protein 1 expression is repressed by miR-155, and its restoration inhibits pancreatic tumor development. Proc Natl Acad Sci U S A.

[4] Chang TC, Wentzel EA, Kent OA, Ramachandran K, Mullendore M, Lee KH (2007). Transactivation of miR-34a by p53 broadly influences gene expression and promotes apoptosis. Mol Cell.

[5] Li N, Fu H, Tie Y, Hu Z, Kong W, Wu Y (2009). miR-34a inhibits migration and invasion by down-regulation of c-Met expression in human hepatocellular carcinoma cells. Cancer letters.

[6] Yamakuchi M, Ferlito M, Lowenstein CJ (2008). miR-34a repression of SIRT1 regulates apoptosis. Proc Natl Acad Sci USA.

[7] Hatziapostolou M, Polytarchou C, Aggelidou E, Drakaki A, Poultsides GA, Jaeger SA (2011). An HNF4alpha-miRNA inflammatory feedback circuit regulates hepatocellular oncogenesis. Cell.

[8] Li KK, Pang JC, Ching AK, Wong CK, Kong X, Wang Y (2009). miR-124 is frequently down-regulated in medulloblastoma and is a negative regulator of SLC16A1. Hum Pathol.

[9] Volinia S, Calin GA, Liu CG, Ambs S, Cimmino A, Petrocca F (2006). A microRNA expression signature of human solid tumors defines cancer gene targets. Proc Natl Acad Sci U S A.

[10] Tian XP, Wang CY, Jin XH, Li M, Wang FW, Huang WJ (2019). Acidic Microenvironment Up-Regulates Exosomal miR-21 and miR-10b in Early-Stage Hepatocellular Carcinoma to Promote Cancer Cell Proliferation and Metastasis. Theranostics.

[11] Li KKW, Pang JC-s, Ching AK-k, Wong CK, Kong X, Wang Y (2009). miR-124 is frequently down-regulated in medulloblastoma and is a negative regulator of SLC16A1. Human pathology.

[12] Bader AG, Brown D, Winkler M (2010). The promise of microRNA replacement therapy. Cancer Res.

[13] Rupaimoole R, Slack FJ (2017). MicroRNA therapeutics: towards a new era for the management of cancer and other diseases. Nat Rev Drug Discov.

[14] Yu AM, Jian C, Yu AH, Tu MJ (2019). RNA therapy: Are we using the right molecules?. Pharmacol Ther.

[15] Costales MG, Childs-Disney JL, Haniff HS, Disney MD (2020). How We Think about Targeting RNA with Small Molecules. J Med Chem.

[16] Yu AM, Choi YH, Tu MJ (2020). RNA drugs and RNA targets for small molecules: Principles, progresses, and challenges. Pharmacol Rev.

[17] Fire A, Xu S, Montgomery MK, Kostas SA, Driver SE, Mello CC (1998). Potent and specific genetic interference by double-stranded RNA in Caenorhabditis elegans. Nature.

[18] Zamore PD, Tuschl T, Sharp PA, Bartel DP (2000). RNAi: double-stranded RNA directs the ATP-dependent cleavage of mRNA at 21 to 23 nucleotide intervals. Cell.

[19] Brodersen P, Sakvarelidze-Achard L, Bruun-Rasmussen M, Dunoyer P, Yamamoto YY, Sieburth L (2008). Widespread translational inhibition by plant miRNAs and siRNAs. Science.

[20] Setten RL, Rossi JJ, Han SP (2019). The current state and future directions of RNAi-based therapeutics. Nat Rev Drug Discov.

[21] FDA. FDA Approves First Drug to Treat Rare Metabolic Disorder. November 23, 2020.

[22] Khvorova A, Watts JK (2017). The chemical evolution of oligonucleotide therapies of clinical utility. Nat Biotechnol.

[23] Beckert B, Masquida B (2011). Synthesis of RNA by *in vitro* transcription. Methods Mol Biol.

[24] Ho PY, Yu AM (2016). Bioengineering of noncoding RNAs for research agents and therapeutics. Wiley Interdiscip Rev RNA.

[25] Burley SK, Berman HM, Bhikadiya C, Bi C, Chen L, Di Costanzo L (2019). RCSB Protein Data Bank: biological macromolecular structures enabling research and education in fundamental biology, biomedicine, biotechnology and energy. Nucleic Acids Res.

[26] Usmani SS, Bedi G, Samuel JS, Singh S, Kalra S, Kumar P (2017). THPdb: Database of FDA-approved peptide and protein therapeutics. PLoS One.

[27] Chen QX, Wang WP, Zeng S, Urayama S, Yu AM (2015). A general approach to high-yield biosynthesis of chimeric RNAs bearing various types of functional small RNAs for broad applications. Nucleic Acids Res.

[28] Ho PY, Duan Z, Batra N, Jilek JL, Tu MJ, Qiu JX (2018). Bioengineered Noncoding RNAs Selectively Change Cellular miRNome Profiles for Cancer Therapy. J Pharmacol Exp Ther.

[29] Ponchon L, Beauvais G, Nonin-Lecomte S, Dardel F (2009). A generic protocol for the expression and purification of recombinant RNA in Escherichia coli using a tRNA scaffold. Nat Protoc.

[30] Wang WP, Ho PY, Chen QX, Addepalli B, Limbach PA, Li MM (2015). Bioengineering Novel Chimeric microRNA-34a for Prodrug Cancer Therapy: High-Yield Expression and Purification, and Structural and Functional Characterization. Pharmacol Exp Ther.

[31] Zhao Y, Tu MJ, Yu YF, Wang WP, Chen QX, Qiu JX (2015). Combination therapy with bioengineered miR-34a prodrug and doxorubicin synergistically suppresses osteosarcoma growth. Biochem Pharmacol.

[32] Jian C, Tu MJ, Ho PY, Duan Z, Zhang Q, Qiu JX (2017). Co-targeting of DNA, RNA, and protein molecules provides optimal outcomes for treating osteosarcoma and pulmonary metastasis in spontaneous and experimental metastasis mouse models. Oncotarget.

[33] Parisien M, Wang X, Pan T (2013). Diversity of human tRNA genes from the 1000-genomes project. RNA Biol.

[34] Novoa EM, Pavon-Eternod M, Pan T, Ribas de Pouplana L (2012). A role for tRNA modifications in genome structure and codon usage. Cell.

[35] Yamakuchi M, Ferlito M, Lowenstein CJ (2008). miR-34a repression of SIRT1 regulates apoptosis. Proc Natl Acad Sci USA.

[36] Li N, Fu H, Tie Y, Hu Z, Kong W, Wu Y (2009). miR-34a inhibits migration and invasion by down-regulation of c-Met expression in human hepatocellular carcinoma cells. Cancer Lett.

[37] He L, He X, Lim LP, de Stanchina E, Xuan Z, Liang Y (2007). A microRNA component of the p53 tumour suppressor network. Nature.

[38] Lin X, Chen W, Wei F, Zhou BP, Hung MC, Xie X (2017). Nanoparticle Delivery of miR-34a Eradicates Long-term-cultured Breast Cancer Stem Cells via Targeting C22ORF28 Directly. Theranostics.

[39] Mohseny AB, Machado I, Cai Y, Schaefer KL, Serra M, Hogendoorn PC (2011). Functional characterization of osteosarcoma cell lines provides representative models to study the human disease. Lab Invest.

[40] Wang Y, Nie H, He X, Liao Z, Zhou Y, Zhou J (2020). The emerging role of super enhancer-derived noncoding RNAs in human cancer. Theranostics.

[41] van Zandwijk N, Pavlakis N, Kao SC, Linton A, Boyer MJ, Clarke S (2017). Safety and activity of microRNA-loaded minicells in patients with recurrent malignant pleural mesothelioma: a first-in-man, phase 1, open-label, dose-escalation study. Lancet Oncol.

[42] Hong DS, Kang YK, Borad M, Sachdev J, Ejadi S, Lim HY (2020). Phase 1 study of MRX34, a liposomal miR-34a mimic, in patients with advanced solid tumours. Br J Cancer.

[43] Rivera-Valentin RK, Zhu L, Hughes DP (2015). Bone sarcomas in pediatrics: progress in our understanding of tumor biology and implications for therapy. Pediatric Drugs.

[44] Bacci G, Rocca M, Salone M, Balladelli A, Ferrari S, Palmerini E (2008). High grade osteosarcoma of the extremities with lung metastases at presentation: treatment with neoadjuvant chemotherapy and simultaneous resection of primary and metastatic lesions. J Surg Oncol.

[45] Zhang K, Dong C, Chen M, Yang T, Wang X, Gao Y (2020). Extracellular vesicle-mediated delivery of miR-101 inhibits lung metastasis in osteosarcoma. Theranostics.

[46] Chang L, Shrestha S, LaChaud G, Scott MA, James AW (2015). Review of microRNA in osteosarcoma and chondrosarcoma. Med Oncol.

