# Supplementary material for: In vivo fermentation production of humanized noncoding RNAs carrying payload miRNAs for targeted anticancer therapy
Source: Theranostics. 2021 Mar 4;11(10):4858–71. doi: 10.7150/thno.56596 (PMC7978307; doi:10.7150/thno.56596)
Supplement: Supplementary file 1 — Supplementary figure and tables. [file thnov11p4858s1.pdf]

Supplementary Materials for

In vivo fermentation production of humanized noncoding RNAs carrying payload miRNAs for targeted anticancer therapy

Peng-Cheng Li[1,2,#], Mei-Juan Tu[2,#], Pui Yan Ho[2,#], Neelu Batra[2], Michelle M.L. Tran[2], Jing-Xin Qiu[3], Theodore Wun[4], Primo N. Lara[5], Xiang Hu[1], Ai-Xi Yu[1,*], Ai-Ming Yu[2,*]

The PDF file includes:

Table S1. Sequences of target recombinant ncRNAs.

Table S2. Primers used for cloning ncRNA expression plasmids.

Figure S1. Body weights of animals during the therapy study.

Table S1. Sequences of humanized recombinant ncRNAs bearing RNAi warheads. Underlined, tRNA; Red and green: payload miRNA or siRNA sequence and complementary sequence, respectively; orange, aptamer.

Target ncRNA	# nts	Sequence (5' to 3')	MW (Da)
**htRNA/pre-miR-34a**			
htRNA^{Lys}/pre-miR-34a	182	GCCUGGAUAGCUCAGUUGGUAGAGCAUCAGACUGGCCAGCU<br>GUGAGUGUUUCUUUGGCAGUGUCUUAGCUGGUUGUUGUGAG<br>CAAUAGUAAGGAAGCAAUCAGCAAGUAUACUGCCCUAGAAGU<br>GCUGCACGUUGUUGGCCCAAUCUGAGGGUCCAGGGUUCAAG<br>UCCCUGUUCAGGCGCCA	58,889
htRNA^{Gln}/pre-miR-34a	181	GGUCCCAUGGUGUAAUGGUUAGCACUCUGGACUGGCCAGCU<br>GUGAGUGUUUCUUUGGCAGUGUCUUAGCUGGUUGUUGUGAG<br>CAAUAGUAAGGAAGCAAUCAGCAAGUAUACUGCCCUAGAAGU<br>GCUGCACGUUGUUGGCCCAAUCCAGCGAUCCGAGUUCAAAUC<br>UCGGUGGGACCUCCA	58,465
htRNA^{Cys}/pre-miR-34a	181	GGGGGCAUAGCUCAGUGGUAGAGCAUUUGACUGGCCAGCUG<br>UGAGUGUUUCUUUGGCAGUGUCUUAGCUGGUUGUUGUGAGC<br>AAUAGUAAGGAAGCAAUCAGCAAGUAUACUGCCCUAGAAGUG<br>CUGCACGUUGUUGGCCCGAUCAAGAGGUCCCUGGUUCAAAU<br>CCAGGUGCCCCCUCCA	58,543
htRNA^{Tyr}/pre-miR-34a	182	CCUUCGAUAGCUCAGUUGGUAGAGCGGAGGACUGGCCAGCU<br>GUGAGUGUUUCUUUGGCAGUGUCUUAGCUGGUUGUUGUGAG<br>CAAUAGUAAGGAAGCAAUCAGCAAGUAUACUGCCCUAGAAGU<br>GCUGCACGUUGUUGGCCCGAUCCUUAGGUCGCUGGUUCGAA<br>UCCGGCUCGAAGGACCA	58,905
htRNA^{Ser}/pre-miR-34a	191	GCAGCGAUGGCCGAGUGGUUAAGGCGUUGGACUGGCCAGCU<br>GUGAGUGUUUCUUUGGCAGUGUCUUAGCUGGUUGUUGUGAG<br>CAAUAGUAAGGAAGCAAUCAGCAAGUAUACUGCCCUAGAAGU<br>GCUGCACGUUGUUGGCCCAAUCCAAUGGGGUCUCCCCGCGC<br>AGGUUCGAACCCUGCUCGCUGCGCCA	61,787
htRNA^{Leu}/pre-miR-34a	192	ACCAGGAUGGCCGAGUGGUUAAGGCGUUGGACUGGCCAGCU<br>GUGAGUGUUUCUUUGGCAGUGUCUUAGCUGGUUGUUGUGAG<br>CAAUAGUAAGGAAGCAAUCAGCAAGUAUACUGCCCUAGAAGU<br>GCUGCACGUUGUUGGCCCGAUCCAAUGGACAUAUGUCCGCG<br>UGGGUUCGAACCCCACUCCUGGUACCA	62,110
**htRNA^{Leu}/pre-miR-34a**			
htRNA^{Leu}/sephadex-aptamer	119	ACCAGGAUGGCCGAGUGGUUAAGGCGUUGGACUAGUAAUUU<br>ACGUCGACGGUGACGUCGAUGGUUGCGGGAUCCAAUGGACA<br>UAUGUCCGCGUGGGUUCGAACCCCACUCCUGGUACCA	38,625
htRNA^{Leu}/pre-miR-34a/miR-200b-3p	192	ACCAGGAUGGCCGAGUGGUUAAGGCGUUGGACUGGCCAGCU<br>GUGAGUGUUUCUUUAAUACUGCCUGGUAAUGAUGAUGUGAG<br>CAAUAGUAAGGAAUCAUCAUUAUAGUGCAGUAUUCUAGAAGU<br>GCUGCACGUUGUUGGCCCGAUCCAAUGGACAUAUGUCCGCG<br>UGGGUUCGAACCCCACUCCUGGUACCA	62,049
htRNA^{Leu}/pre-miR-34a/miR-148-3p	192	ACCAGGAUGGCCGAGUGGUUAAGGCGUUGGACUGGCCAGCU<br>GUGAGUGUUUCUUUCAGUGCACUACAGAACUUUGUUGUGAG<br>CAAUAGUAAGGAAGCAAAGUUCGUAUGUGCACUGCUAGAAGU<br>GCUGCACGUUGUUGGCCCGAUCCAAUGGACAUAUGUCCGCG<br>UGGGUUCGAACCCCACUCCUGGUACCA	62,078
htRNA^{Leu}/pre-miR-34a/miR-22-3p	192	ACCAGGAUGGCCGAGUGGUUAAGGCGUUGGACUGGCCAGCU<br>GUGAGUGUUUCUUAAGCUGCCAGUUGAAGAACUGUUGUGAG<br>CAAUAGUAAGGAAGCAGUUCUUAGCUUGGCAGCUCUAGAAGU<br>GCUGCACGUUGUUGGCCCGAUCCAAUGGACAUAUGUCCGCG<br>UGGGUUCGAACCCCACUCCUGGUACCA	62,133
htRNA^{Leu}/pre-miR-34a/miR-6775-3p	192	ACCAGGAUGGCCGAGUGGUUAAGGCGUUGGACUGGCCAGCU<br>GUGAGUGUUUCUUAGGCCCUGUCCUCUGCCCCAGUUGUGAG<br>CAAUAGUAAGGAAGCUGGGGCAAGGCGCAGGGCCCUAGAAG	62,167

		UGCUGCACGUUGUUGGCCCGAUCCAAUGGACAUAUGUCCGC<br>GUGGGUUCGAACCCCACUCCUGGUACCA	
htRNA^{Leu}/pre-miR-34a/miR-133a-3p	193	ACCAGGAUGGCCGAGUGGUUAAGGCGUUGGACUGGCCAGCU<br>GUGAGUGUUUCUUUUUGGUCCCCUUCAACCAGCUGUGUGAG<br>CAAUAGUAAGGAAGCAGCUGGUUAAGUGGGACCAACUAGAAG<br>UGCUGCACGUUGUUGGCCCGAUCCAAUGGACAUAUGUCCGC<br>GUGGGUUCGAACCCCACUCCUGGUACCA	62,413
htRNA^{Leu}/pre-miR-34a/miR-124-3p	192	ACCAGGAUGGCCGAGUGGUUAAGGCGUUGGACUGGCCAGCU<br>GUGAGUGUUUCUUUAAGGCACGCGGUGAAUGCCGUUGUGAG<br>CAAUAGUAAGGAAGCGGUGUUCCCGUCGUGCCUUCUAGAAG<br>UGCUGCACGUUGUUGGCCCGAUCCAAUGGACAUAUGUCCGC<br>GUGGGUUCGAACCCCACUCCUGGUACCA	62,132
htRNA^{Leu}/pre-miR-34a/let-7c-5p	193	ACCAGGAUGGCCGAGUGGUUAAGGCGUUGGACUGGCCAGCU<br>GUGAGUGUUUCUUUGAGGUAGUAGGUUGUAUGGUUUGUGAG<br>CAAUAGUAAGGAAGAACUGUACACCUUACUACCUUUCAGAAG<br>UGCUGCACGUUGUUGGCCCGAUCCAAUGGACAUAUGUCCGC<br>GUGGGUUCGAACCCCACUCCUGGUACCA	62,402
htRNA^{Leu}/pre-miR-34a/NRF2-siRNA	192	ACCAGGAUGGCCGAGUGGUUAAGGCGUUGGACUGGCCAGCU<br>GUGAGUGUUUCUUUAAUUGUCAACUACUGUCAGUUUGUGAG<br>CAAUAGUAAGGAAAACUGACAGAGUAUGACAAUUCUAGAAGU<br>GCUGCACGUUGUUGGCCCGAUCCAAUGGACAUAUGUCCGCG<br>UGGGUUCGAACCCCACUCCUGGUACCA	62,055
htRNA^{Leu}/pre-miR-34a/miR-122-5p	193	ACCAGGAUGGCCGAGUGGUUAAGGCGUUGGACUGGCCAGCU<br>GUGAGUGUUUCUUUGGAGUGUGACAAUGGUGUUUGUGUGAG<br>CAAUAGUAAGGAAGCAAACGCCAUGUACACACUCCCUAGAAG<br>UGCUGCACGUUGUUGGCCCGAUCCAAUGGACAUAUGUCCGC<br>GUGGGUUCGAACCCCACUCCUGGUACCA	62,461
htRNA^{Leu}/pre-miR-34a/miR-328-3p	192	ACCAGGAUGGCCGAGUGGUUAAGGCGUUGGACUGGCCAGCU<br>GUGAGUGUUUCUUCUGGCCCUCUCUGCCCUUCCGUUGUGAG<br>CAAUAGUAAGGAAGCGGGGGGGAGAUGGGGGCCAUUAGAAG<br>UGCUGCACGUUGUUGGCCCGAUCCAAUGGACAUAUGUCCGC<br>GUGGGUUCGAACCCCACUCCUGGUACCA	62,202
htRNA^{Leu}/pre-miR-34a/miR-7-1-5p	192	ACCAGGAUGGCCGAGUGGUUAAGGCGUUGGACUGGCCAGCU<br>GUGAGUGUUUCUUUGGAAGACUAGUGAUUUUGUUGUGUGAG<br>CAAUAGUAAGGAACAACAAAAUACUCAGUCUUCCCUAGAAGU<br>GCUGCACGUUGUUGGCCCGAUCCAAUGGACAUAUGUCCGCG<br>UGGGUUCGAACCCCACUCCUGGUACCA	62,047
htRNA^{Leu}/pre-miR-34a/miR-92a-3p	192	ACCAGGAUGGCCGAGUGGUUAAGGCGUUGGACUGGCCAGCU<br>GUGAGUGUUUCUUUAUUGCACUUGUCCCGGCCUGUUGUGAG<br>CAAUAGUAAGGAAACAGGCCGGACAUAGUGCAAUCUAGAAGU<br>GCUGCACGUUGUUGGCCCGAUCCAAUGGACAUAUGUCCGCG<br>UGGGUUCGAACCCCACUCCUGGUACCA	62,068
htRNA^{Leu}/pre-miR-34a/miR-126-3p	192	ACCAGGAUGGCCGAGUGGUUAAGGCGUUGGACUGGCCAGCU<br>GUGAGUGUUUCUUUCGUACCGUGAGUAAUAAUGCGUGUGAG<br>CAAUAGUAAGGAAUGCAUUAUUCUCUAUGGUACGCUAGAAGU<br>GCUGCACGUUGUUGGCCCGAUCCAAUGGACAUAUGUCCGCG<br>UGGGUUCGAACCCCACUCCUGGUACCA	62,056
htRNA^{Leu}/pre-miR-34a/miR-132-3p	192	ACCAGGAUGGCCGAGUGGUUAAGGCGUUGGACUGGCCAGCU<br>GUGAGUGUUUCUUUAACAGUCUACAGCCAUGGUCGUGUGAG<br>CAAUAGUAAGGAACGACCGUGGUGUCAGAUUGUUCUAGAAGU<br>GCUGCACGUUGUUGGCCCGAUCCAAUGGACAUAUGUCCGCG<br>UGGGUUCGAACCCCACUCCUGGUACCA	62,109
htRNA^{Leu}/pre-miR-34a/miR-137-3p	192	ACCAGGAUGGCCGAGUGGUUAAGGCGUUGGACUGGCCAGCU<br>GUGAGUGUUUCUUUUAUUGCUUAAGAAUACGCGUAGGUGAG<br>CAAUAGUAAGGACUACGCGUAUCUUCGGGUGGUACUAGAAGU<br>GCUGCACGUUGUUGGCCCGAUCCAAUGGACAUAUGUCCGCG<br>UGGGUUCGAACCCCACUCCUGGUACCA	62,111
htRNA^{Leu}/pre-miR-34a/miR-140-5p	192	ACCAGGAUGGCCGAGUGGUUAAGGCGUUGGACUGGCCAGCU<br>GUGAGUGUUUCUUCAGUGGUUUUACCCUAUGGUAGUGUGAG<br>CAAUAGUAAGGAACUACCAUAGGUAUAAACCACUCUAGAAGU<br>GCUGCACGUUGUUGGCCCGAUCCAAUGGACAUAUGUCCGCG<br>UGGGUUCGAACCCCACUCCUGGUACCA	62,022

htRNA^{Leu}/pre-miR-34a/miR-141-3p	192	ACCAGGAUGGCCGAGUGGUUAAGGCGUUGGACUGGCCAGCU<br>GUGAGUGUUUCUUUAACACUGUCUGGUAAAGAUGGUGUGAG<br>CAAUAGUAAGGAACCAUCUUUACAGUACAGUGUUCUAGAAGU<br>GCUGCACGUUGUUGGCCCGAUCCAAUGGACAUAUGUCCGCG<br>UGGGUUCGAACCCCACUCCUGGUACCA	62,063
htRNA^{Leu}/pre-miR-34a/miR-142-3p	194	ACCAGGAUGGCCGAGUGGUUAAGGCGUUGGACUGGCCAGCU<br>GUGAGUGUUUCUUUGUAGUGUUUCCUACUUUAUGGAUGUGA<br>GCAAUAGUAAGGAAUCCAUAAAGUGGACAGCACUACUCAGAA<br>GUGCUGCACGUUGUUGGCCCGAUCCAAUGGACAUAUGUCCG<br>CGUGGGUUCGAACCCCACUCCUGGUACCA	62,674
htRNA^{Leu}/pre-miR-34a/miR-194-3p	192	ACCAGGAUGGCCGAGUGGUUAAGGCGUUGGACUGGCCAGCU<br>GUGAGUGUUUCUUUGUAACAGCAACUCCAUGUGGAUGUGAG<br>CAAUAGUAAGGAAUCCACGUGGGUUAGCUGUUACUUAGAAGU<br>GCUGCACGUUGUUGGCCCGAUCCAAUGGACAUAUGUCCGCG<br>UGGGUUCGAACCCCACUCCUGGUACCA	62,094
htRNA^{Leu}/pre-miR-34a/miR-205-5p	192	ACCAGGAUGGCCGAGUGGUUAAGGCGUUGGACUGGCCAGCU<br>GUGAGUGUUUCUUUCCUUCAUUCCACCGGAGUCUGUGUGAG<br>CAAUAGUAAGGAACAGACUCCGUGGUAAUGAAGGCUAGAAGU<br>GCUGCACGUUGUUGGCCCGAUCCAAUGGACAUAUGUCCGCG<br>UGGGUUCGAACCCCACUCCUGGUACCA	62,068
**htRNA^{Ser}/pre-miR-34a**			
htRNA^{Ser}/sephadex-aptamer	118	GCAGCGAUGGCCGAGUGGUUAAGGCGUUGGACUAGUAAUUU<br>ACGUCGACGGUGACGUCGAUGGUUGCGGAAUCCAAUGGGGU<br>CUCCCCGCGCAGGUUCGAACCCUGCUCGCUGCGCCA	38,302
htRNA^{Ser}/pre-miR-34a/BCL2-siRNA	191	GCAGCGAUGGCCGAGUGGUUAAGGCGUUGGACUGGCCAGCU<br>GUGAGUGUUUCUUUACAGUUCCACAAAGGCAUCCUUGUGAGC<br>AAUAGUAAGGAAGGGAUGCCUUGUGGGAACUGUCUAGAAGU<br>GCUGCACGUUGUUGGCCCAAUCCAAUGGGGUCUCCCCGCGC<br>AGGUUCGAACCCUGCUCGCUGCGCCA	61,809
htRNA^{Ser}/pre-miR-34a/AGR2-siRNA	191	GCAGCGAUGGCCGAGUGGUUAAGGCGUUGGACUGGCCAGCU<br>GUGAGUGUUUCUUUUAAAGCUUGACUGUGUGGGCUUGUGAG<br>CAAUAGUAAGGAAGGCCCACACGUCUAAGCUUUACUAGAAGU<br>GCUGCACGUUGUUGGCCCAAUCCAAUGGGGUCUCCCCGCGC<br>AGGUUCGAACCCUGCUCGCUGCGCCA	61,747
htRNA^{Ser}/pre-miR-34a/miR-124-3p	191	GCAGCGAUGGCCGAGUGGUUAAGGCGUUGGACUGGCCAGCU<br>GUGAGUGUUUCUUUAAGGCACGCGGUGAAUGCCGUUGUGAG<br>CAAUAGUAAGGAAGCGGUGUUCCCGUCGUGCCUUCUAGAAG<br>UGCUGCACGUUGUUGGCCCAAUCCAAUGGGGUCUCCCCGCG<br>CAGGUUCGAACCCUGCUCGCUGCGCCA	61,809
htRNA^{Ser}/pre-miR-34a/let-7c-5p	192	GCAGCGAUGGCCGAGUGGUUAAGGCGUUGGACUGGCCAGCU<br>GUGAGUGUUUCUUUGAGGUAGUAGGUUGUAUGGUUUGUGAG<br>CAAUAGUAAGGAAGAACUGUACACCUUACUACCUUUCAGAAG<br>UGCUGCACGUUGUUGGCCCAAUCCAAUGGGGUCUCCCCGCG<br>CAGGUUCGAACCCUGCUCGCUGCGCCA	62,079
htRNA^{Ser}/pre-miR-34a/miR-328-3p	191	GCAGCGAUGGCCGAGUGGUUAAGGCGUUGGACUGGCCAGCU<br>GUGAGUGUUUCUUCUGGCCCUCUCUGCCCUUCCGUUGUGAG<br>CAAUAGUAAGGAAGCGGGGGGAGAUGGGGGCCAUUAGAAG<br>UGCUGCACGUUGUUGGCCCAAUCCAAUGGGGUCUCCCCGCG<br>CAGGUUCGAACCCUGCUCGCUGCGCCA	61,879
htRNA^{Ser}/pre-miR-34a/miR-200b-3p	191	GCAGCGAUGGCCGAGUGGUUAAGGCGUUGGACUGGCCAGCU<br>GUGAGUGUUUCUUUAAUACUGCCUGGUAAUGAUGAUGUGAG<br>CAAUAGUAAGGAAUCAUCAUUAUAGUGCAGUAUUCUAGAAGU<br>GCUGCACGUUGUUGGCCCAAUCCAAUGGGGUCUCCCCGCGC<br>AGGUUCGAACCCUGCUCGCUGCGCCA	61,726
htRNA^{Ser}/pre-miR-34a/miR-22-3p	191	GCAGCGAUGGCCGAGUGGUUAAGGCGUUGGACUGGCCAGCU<br>GUGAGUGUUUCUUAAGCUGCCAGUUGAAGAACUGUUGUGAG<br>CAAUAGUAAGGAAGCAGUUCUUAGCUUGGCAGCUCUAGAAGU<br>GCUGCACGUUGUUGGCCCAAUCCAAUGGGGUCUCCCCGCGC<br>AGGUUCGAACCCUGCUCGCUGCGCCA	61,810
htRNA^{Ser}/pre-miR-34a/miR-148-3p	191	GCAGCGAUGGCCGAGUGGUUAAGGCGUUGGACUGGCCAGCU<br>GUGAGUGUUUCUUUCAGUGCACUACAGAACUUUGUUGUGAG	61,755

		CAAUAGUAAGGAAGCAAAGUUCGUAUGUGCACUGCUAGAAGU GCUGCACGUUGUUGGCCCAAUCCAAUGGGGUCUCCCCGCGC AGGUUCGAACCCUGCUCGCUGCGCCA	
htRNA^{Ser}/pre-miR-34a/miR-6775-3p	191	GCAGCGAUGGCCGAGUGGUUAAGGCGUUGGACUGGCCAGCU GUGAGUGUUUCUUAGGCCCUGUCCUCUGCCCCAGUUGUGAG CAAUAGUAAGGAAGCUGGGGCAAGGCGCAGGGCCCUAGAAG UGCUGCACGUUGUUGGCCCAAUCCAAUGGGGUCUCCCCGCG CAGGUUCGAACCCUGCUCGCUGCGCCA	61,844
htRNA^{Ser}/pre-miR-34a/NRF2-siRNA	191	GCAGCGAUGGCCGAGUGGUUAAGGCGUUGGACUGGCCAGCU GUGAGUGUUUCUUUAAUUGUCAACUACUGUCAGUUUGUGAG CAAUAGUAAGGAAAACUGACAGAGUAUGACAAUUCUAGAAGU GCUGCACGUUGUUGGCCCAAUCCAAUGGGGUCUCCCCGCGC AGGUUCGAACCCUGCUCGCUGCGCCA	61,732

Table S2. Primers used for the construction of RNA expression plasmids.

Target ncRNA	Cloning Primers (5' to 3')	
**htRNA/pre-miR-34a**		
htRNA^{Lys}/pre-miR-34a	F	TTGTAACGCTGAATTCGCCTGGATAGCTCAGTTGGTAGAGCATCAGACTGGCCAGCTGTGAGTG
	R	CTTTCGCTAAGGATCTGCAGTGGGCGCCTGAACAGGGACTTGAACCCTGGACCCTCAGATTGGGCCAACAACGTGC
htRNA^{Gln}/pre-miR-34a	F	TTGTAACGCTGAATTCGGTCCCATGGTGTAATGGTTAGCACTCTGGACTGGCCAGCTGTGAGTG
	R	CTTTCGCTAAGGATCTGCAGTGGAGGTCCCACCGAGATTTGAACTCGGATCGCTGGATTGGGCCAACAACGTGC
htRNA^{Cys}/pre-miR-34a	F	TTGTAACGCTGAATTCGGGGGCATAGCTCAGTGGTAGAGCATTTGACTGGCCAGCTGTGAGTG
	R	CTTTCGCTAAGGATCTGCAGTGGAGGGGGCACCTGGATTTGAACCAGGGACCTCTTGATCGGGCCAACAACGTGC
htRNA^{Tyr}/pre-miR-34a	F	TTGTAACGCTGAATTCCCTTCGATAGCTCAGTTGGTAGAGCGGAGGACTGGCCAGCTGTGAGTG
	R	CTTTCGCTAAGGATCTGCAGTGGTCCTTCGAGCCGGATTCGAACCAGCGACCTAAGGATCGGGCCAACAACGTGC
htRNA^{Ser}/pre-miR-34a	F	TTGTAACGCTGAATTCGCAGCGATGGCCGAGTGGTTAAGGCGTTGGACTGGCCAGCTGTGAGTG
	R	CTTTCGCTAAGGATCTGCAGTGGCGCAGCGAGCAGGGTTCGAACCTGCGCGGGGAGACCCCATTGGATTGGGCCAACAACGTGC
htRNA^{Leu}/pre-miR-34a	F	TTGTAACGCTGAATTCACCAGGATGGCCGAGTGGTTAAGGCGTTGGACTGGCCAGCTGTGAGTG
	R	CTTTCGCTAAGGATCTGCAGTGGTACCAGGAGTGGGGTTCGAACCCACGCGGACATATGTCCATTGGATCGGGCCAACAACGTGC
**htRNA^{Leu}/pre-miR-34a**		
htRNA^{Leu}/sephadex-aptamer	F	TTGTAACGCTGAATTCACCAGGATGGCCGAGTGGTTAAGGCGTTGGACTAGTAATTTACGTCGACGGTGACGTCGATGGTTGCG
	R	CTTTCGCTAAGGATCTGCAGTGGTACCAGGAGTGGGGTTCGAACCCACGCGGACATATGTCCATTGGATCCCGCAACCATCGACGTCAC
htRNA^{Leu}/pre-miR-34a/miR-200b-3p	F	TTGTAACGCTGAATTCACCAGGATGGCCGAGTGGTTAAGGCGTTGGACTGGCCAGCTGTGAGTGTTTCTTTAATACTGCCTGGTAATGATGATGTGAGCAATAGTAAGGAATCATCA
	R	CTTTCGCTAAGGATCTGCAGTGGTACCAGGAGTGGGGTTCGAACCCACGCGGACATATGTCCATTGGATCGGGCCAACAACGTGCAGCACTTCTAGAATACTGCACTATAATGATGATTCCTTACT
htRNA^{Leu}/pre-miR-34a/miR-148-3p	F	TTGTAACGCTGAATTCACCAGGATGGCCGAGTGGTTAAGGCGTTGGACTGGCCAGCTGTGAGTGTTTCTTTCAGTGCACTACAGAACTTTGTTGTGAGCAATAGTAAGGAAGCAAAGTTC
	R	CTTTCGCTAAGGATCTGCAGTGGTACCAGGAGTGGGGTTCGAACCCACGCGGACATATGTCCATTGGATCGGGCCAACAACGTGCAGCACTTCTAGCAGTGCACATACGAACTTTGCTTCCTT
htRNA^{Leu}/pre-miR-34a/miR-22-3p	F	TTGTAACGCTGAATTCACCAGGATGGCCGAGTGGTTAAGGCGTTGGACTGGCCAGCTGTGAGTGTTTCTTAAGCTGCCAGTTGAAGAACTGTTGTGAGCAATAGTAAGGAAGTAGTTCTT
	R	CTTTCGCTAAGGATCTGCAGTGGTACCAGGAGTGGGGTTCGAACCCACGCGGACATATGTCCATTGGATCGGGCCAACAACGTGCAGCACTTCTAGAGCTGCCAAGCTAAGAACTACTTCCTTAC
htRNA^{Leu}/pre-miR-34a/miR-6775-3p	F	TTGTAACGCTGAATTCACCAGGATGGCCGAGTGGTTAAGGCGTTGGACTGGCCAGCTGTGAGTGTTTCTTAGGCCCTGTCCTCTGCCCCAGTTGTGAGCAATAGTAAGGAAGCTGGGGC
	R	CTTTCGCTAAGGATCTGCAGTGGTACCAGGAGTGGGGTTCGAACCCACGCGGACATATGTCCATTGGATCGGGCCAACAACGTGCAGCACTTCTAGGGCCCTGCGCCTTGCCCCAGCTTCCTTA
htRNA^{Leu}/pre-miR-34a/miR-133a-3p	F	TTGTAACGCTGAATTCACCAGGATGGCCGAGTGGTTAAGGCGTTGGACTGGCCAGCTGTGAGTGTTTCTTTTTGGTCCCCTTCAACCAGCTGTGTGAGCAATAG

	R	CTTTCGCTAAGGATCTGCAGTGGTACCAGGAGTGGGGTTCGAACCCACGCGGACATATGTCCATTGGATCGGGCCAACAACGTGCAGCACTTCTAGTTGGTCCCACTTAACCAGCTGCTTCCTTACTAT
htRNA^{Leu}/pre-miR-34a/miR-124-3p	F	uses the same primer as in htRNA^{Leu}/pre-miR-34a plus corresponding btRNA/hsa-miR-34a plasmid as template
	R	uses the same primer as in htRNA^{Leu}/pre-miR-34a plus corresponding btRNA/hsa-miR-34a plasmid as template
htRNA^{Leu}/pre-miR-34a/let-7c-5p	F	uses the same primer as in htRNA^{Leu}/pre-miR-34a plus corresponding btRNA/hsa-miR-34a plasmid as template
	R	uses the same primer as in htRNA^{Leu}/pre-miR-34a plus corresponding btRNA/hsa-miR-34a plasmid as template
htRNA^{Leu}/pre-miR-34a/NRF2-siRNA	F	uses the same primer as in htRNA^{Leu}/pre-miR-34a plus corresponding btRNA/hsa-miR-34a plasmid as template
	R	uses the same primer as in htRNA^{Leu}/pre-miR-34a plus corresponding btRNA/hsa-miR-34a plasmid as template
htRNA^{Leu}/pre-miR-34a/miR-122-5p	F	uses the same primer as in htRNA^{Leu}/pre-miR-34a plus corresponding btRNA/hsa-miR-34a plasmid as template
	R	uses the same primer as in htRNA^{Leu}/pre-miR-34a plus corresponding btRNA/hsa-miR-34a plasmid as template
htRNA^{Leu}/pre-miR-34a/miR-328-3p	F	uses the same primer as in htRNA^{Leu}/pre-miR-34a plus corresponding btRNA/hsa-miR-34a plasmid as template
	R	uses the same primer as in htRNA^{Leu}/pre-miR-34a plus corresponding btRNA/hsa-miR-34a plasmid as template
htRNA^{Leu}/pre-miR-34a/miR-7-1-5p	F	TTGTAACGCTGAATTCACCAGGATGGCCGAGTGGTTAAGGCGTTGGACTGGCCAGCTGTGAGTGTTTCTTTGGAAGACTAGTGATTTTGTTGTGTGAGCAATAGTAAGGAACAACAAAAT
	R	CTTTCGCTAAGGATCTGCAGTGGTACCAGGAGTGGGGTTCGAACCCACGCGGACATATGTCCATTGGATCGGGCCAACAACGTGCAGCACTTCTAGGGAAGACTGAGTATTTTGTTGTTCCTTA
htRNA^{Leu}/pre-miR-34a/miR-92a-3p	F	TTGTAACGCTGAATTCACCAGGATGGCCGAGTGGTTAAGGCGTTGGACTGGCCAGCTGTGAGTGTTTCTTTATTGCACTTGTCCCGGCCTGTTGTGAGCAATAGTAAGGAAACAGGCCGG
	R	CTTTCGCTAAGGATCTGCAGTGGTACCAGGAGTGGGGTTCGAACCCACGCGGACATATGTCCATTGGATCGGGCCAACAACGTGCAGCACTTCTAGATTGCACTATGTCCGGCCTGTTTCCTT
htRNA^{Leu}/pre-miR-34a/miR-126-3p	F	TTGTAACGCTGAATTCACCAGGATGGCCGAGTGGTTAAGGCGTTGGACTGGCCAGCTGTGAGTGTTTCTTTCGTACCGTGAGTAATAATGCGTGTGAGCAATAGTAAGGAATGCATTATT
	R	CTTTCGCTAAGGATCTGCAGTGGTACCAGGAGTGGGGTTCGAACCCACGCGGACATATGTCCATTGGATCGGGCCAACAACGTGCAGCACTTCTAGCGTACCATAGAGAATAATGCATTCCTT
htRNA^{Leu}/pre-miR-34a/miR-132-3p	F	TTGTAACGCTGAATTCACCAGGATGGCCGAGTGGTTAAGGCGTTGGACTGGCCAGCTGTGAGTGTTTCTTTAACAGTCTACAGCCATGGTCGTGTGAGCAATAGTAAGGAACGACCGTGG
	R	CTTTCGCTAAGGATCTGCAGTGGTACCAGGAGTGGGGTTCGAACCCACGCGGACATATGTCCATTGGATCGGGCCAACAACGTGCAGCACTTCTAGAACAATCTGACACCACGGTCGTTCCTT
htRNA^{Leu}/pre-miR-34a/miR-137-3p	F	TTGTAACGCTGAATTCACCAGGATGGCCGAGTGGTTAAGGCGTTGGACTGGCCAGCTGTGAGTGTTTCTTTTATTGCTTAAGAATACGCGTAGGTGAGCAATAGTAAGGACTACGCGTA
	R	CTTTCGCTAAGGATCTGCAGTGGTACCAGGAGTGGGGTTCGAACCCACGCGGACATATGTCCATTGGATCGGGCCAACAACGTGCAGCACTTCTAGTACCACCCGAAGATACGCGTAGTCCTTACT
htRNA^{Leu}/pre-miR-34a/miR-140-5p	F	TTGTAACGCTGAATTCACCAGGATGGCCGAGTGGTTAAGGCGTTGGACTGGCCAGCTGTGAGTGTTTCTTCAGTGGTTTTACCCTATGGTAGTGTGAGCAATAGTAAGGAACTACCATA
	R	CTTTCGCTAAGGATCTGCAGTGGTACCAGGAGTGGGGTTCGAACCCACGCGGACATATGTCCATTGGATCGGGCCAACAACGTGCAGCACTTCTAGAGTGGTTCGTACCTATGGTAGTTCCTT
htRNA^{Leu}/pre-miR-34a/miR-141-3p	F	TTGTAACGCTGAATTCACCAGGATGGCCGAGTGGTTAAGGCGTTGGACTGGCCAGCTGTGAGTGTTTCTTTAACACTGTCTGGTAAAGATGGTGTGAGCAATAGTAAGGAACCATCTTTA

	R	CTTTCGCTAAGGATCTGCAGTGGTACCAGGAGTGGGGTTCGAACCCACGCGGACATATGTCCATTGGATCGGGCCAACAACGTGCAGCACTTCTAGAACACTGTACTGTAAAGATGGTTCCTT
htRNA^{Leu}/pre-miR-34a/miR-142-3p	F	TTGTAACGCTGAATTCACCAGGATGGCCGAGTGGTTAAGGCGTTGGACTGGCCAGCTGTGAGTGTTTCTTTGTAGTGTTTCCTACTTTATGGATGTGAGCAATAGTAAGGAATCCATAAAG
	R	CTTTCGCTAAGGATCTGCAGTGGTACCAGGAGTGGGGTTCGAACCCACGCGGACATATGTCCATTGGATCGGGCCAACAACGTGCAGCACTTCTGAGTAGTGCTGTCCACTTTATGGATTCCTT
htRNA^{Leu}/pre-miR-34a/miR-194-3p	F	TTGTAACGCTGAATTCACCAGGATGGCCGAGTGGTTAAGGCGTTGGACTGGCCAGCTGTGAGTGTTTCTTTGTAACAGCAACTCCATGTGGATGTGAGCAATAGTAAGGAATCCACGTG
	R	CTTTCGCTAAGGATCTGCAGTGGTACCAGGAGTGGGGTTCGAACCCACGCGGACATATGTCCATTGGATCGGGCCAACAACGTGCAGCACTTCTAAGTAACAGCTAACCCACGTGGATTCCTTA
htRNA^{Leu}/pre-miR-34a/miR-205-5p	F	TTGTAACGCTGAATTCACCAGGATGGCCGAGTGGTTAAGGCGTTGGACTGGCCAGCTGTGAGTGTTTCTTTCCTTCATTCCACCGGAGTCTGTGTGAGCAATAGTAAGGAACAGACTCCG
	R	CTTTCGCTAAGGATCTGCAGTGGTACCAGGAGTGGGGTTCGAACCCACGCGGACATATGTCCATTGGATCGGGCCAACAACGTGCAGCACTTCTAGCCTTCATTACCACGGAGTCTGTTCCTT
**htRNA^{Ser}/pre-miR-34a**		
htRNA^{Ser}/sephadex-aptamer	F	TTGTAACGCTGAATTCGCAGCGATGGCCGAGTGGTTAAGGCGTTGGACTAGTAATTTACGTCGACGGTGACGTCGATGGTTG
	R	CTTTCGCTAAGGATCTGCAGTGGCGCAGCGAGCAGGGTTCGAACCTGCGCGGGGAGACCCCATTGGATTCCGCAACCATCGACGTC
htRNA^{Ser}/pre-miR-34a/BCL2-siRNA	F	TTGTAACGCTGAATTCGCAGCGATGGCCGAGTGGTTAAGGCGTTGGACTGGCCAGCTGTGAGTGTTTCTTTACAGTTCCACAAAGGCATCCTTGTGAGCAATAGTAAGGAAGGGATGCCTTG
	R	CTTTCGCTAAGGATCTGCAGTGGCGCAGCGAGCAGGGTTCGAACCTGCGCGGGGAGACCCCATTGGATTGGGCCAACAACGTGCAGCACTTCTAGACAGTTCCCACAAGGCATCCCTTCC
htRNA^{Ser}/pre-miR-34a/AGR2-siRNA	F	TTGTAACGCTGAATTCGCAGCGATGGCCGAGTGGTTAAGGCGTTGGACTGGCCAGCTGTGAGTGTTTCTTTTAAAGCTTGACTGTGTGGGCTTGTGAGCAATAGTAAGGAAGGCCCACACGT
	R	CTTTCGCTAAGGATCTGCAGTGGCGCAGCGAGCAGGGTTCGAACCTGCGCGGGGAGACCCCATTGGATTGGGCCAACAACGTGCAGCACTTCTAGTAAAGCTTAGACGTGTGGGCCTTCC
htRNA^{Ser}/pre-miR-34a/miR-124-3p	F	uses the same primer as in htRNA^{Ser}/pre-miR-34a plus corresponding btRNA/hsa-miR-34a plasmid as template
	R	uses the same primer as in htRNA^{Ser}/pre-miR-34a plus corresponding btRNA/hsa-miR-34a plasmid as template
htRNA^{Ser}/pre-miR-34a/let-7c-5p	F	uses the same primer as in htRNA^{Ser}/pre-miR-34a plus corresponding btRNA/hsa-miR-34a plasmid as template
	R	uses the same primer as in htRNA^{Ser}/pre-miR-34a plus corresponding btRNA/hsa-miR-34a plasmid as template
htRNA^{Ser}/pre-miR-34a/miR-328-3p	F	uses the same primer as in htRNA^{Ser}/pre-miR-34a plus corresponding btRNA/hsa-miR-34a plasmid as template
	R	uses the same primer as in htRNA^{Ser}/pre-miR-34a plus corresponding btRNA/hsa-miR-34a plasmid as template
htRNA^{Ser}/pre-miR-34a/miR-200b-3p	F	uses the same primer as in htRNA^{Ser}/pre-miR-34a plus corresponding btRNA/hsa-miR-34a plasmid as template
	R	uses the same primer as in htRNA^{Ser}/pre-miR-34a plus corresponding btRNA/hsa-miR-34a plasmid as template
htRNA^{Ser}/pre-miR-34a/miR-22-3p	F	uses the same primer as in htRNA^{Ser}/pre-miR-34a plus corresponding btRNA/hsa-miR-34a plasmid as template
	R	uses the same primer as in htRNA^{Ser}/pre-miR-34a plus corresponding btRNA/hsa-miR-34a plasmid as template
htRNA^{Ser}/pre-miR-34a/miR-148-3p	F	uses the same primer as in htRNA^{Ser}/pre-miR-34a plus corresponding btRNA/hsa-miR-34a plasmid as template
	R	uses the same primer as in htRNA^{Ser}/pre-miR-34a plus corresponding btRNA/hsa-miR-34a plasmid as template

htRNA^{Ser}/pre-miR-34a/miR-6775-3p	F	uses the same primer as in htRNA^{Ser}/pre-miR-34a plus corresponding btRNA/hsa-miR-34a plasmid as template
	R	uses the same primer as in htRNA^{Ser}/pre-miR-34a plus corresponding btRNA/hsa-miR-34a plasmid as template
htRNA^{Ser}/pre-miR-34a/NRF2-siRNA	F	uses the same primer as in htRNA^{Ser}/pre-miR-34a plus corresponding btRNA/hsa-miR-34a plasmid as template
	R	uses the same primer as in htRNA^{Ser}/pre-miR-34a plus corresponding btRNA/hsa-miR-34a plasmid as template

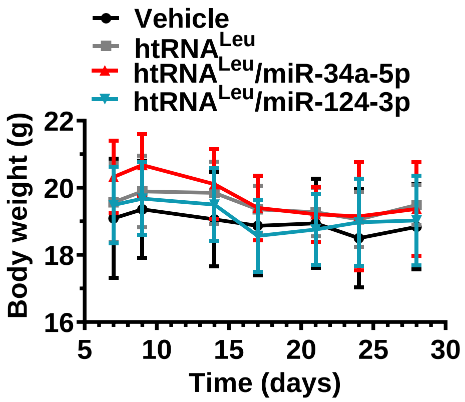

Figure S1. Animal body weights did not differ between different treatment groups (not significant between any groups; 2-way ANOVA with Bonferroni post-tests).
